# Macrophage Plasticity: Phenotypic and Functional Profiles Across Pathological Microenvironments

**DOI:** 10.3390/ijms27125333

**Published:** 2026-06-12

**Authors:** Alessandra Falda

**Affiliations:** Laboratory Medicine Unit, Integrated Diagnostic Services-DIDAS, Padua University Hospital, 35128 Padua, Italy; alessandra.falda@aopd.veneto.it

**Keywords:** macrophages, plasticity, microenvironment, macrophage-reprogramming therapy

## Abstract

Macrophages are highly plastic innate immune cells that adopt context-dependent phenotypes along a continuum, integrating developmental origin with local microenvironmental cues rather than conforming to discrete M1/M2 states. This review delineates the molecular circuits shaping macrophage identity—TLR/cytokine signaling, microRNA networks, metabolic rewiring, and epigenetic mechanisms including histone lactylation—and traces how circulating monocyte subsets contribute to tissue macrophage diversity. We examine macrophage plasticity across a broad disease spectrum—oncology, autoimmune and rheumatic diseases, inflammatory bowel disease, infectious diseases, metabolic disorders, and neurological conditions—showing that the pathogenic phenotype is strikingly context-dependent: for instance, M2-like tumor-associated macrophages promote immune evasion in solid tumors, whereas M1-skewed programs drive tissue damage in autoimmunity. Soluble markers (sCD163, sCD14, soluble mannose receptor) are emerging biomarkers of disease activity and prognosis. High-dimensional flow cytometry and mass cytometry (CyTOF) bridge molecular biology and clinical phenotyping, enabling integrated readouts of surface phenotype, intracellular signaling, and metabolic state. Therapeutic strategies discussed include selective tumor-associated macrophage (TAM) reprogramming, chimeric antigen receptor (CAR)-M cell therapies, and biomaterial-based platforms. Future priorities encompass spatially resolved multi-omics, epigenetic and metabolic targeting, and macrophage-centered vaccine approaches. Standardized cytometry panels will be essential for biomarker-guided stratification and context-specific interventions.

## 1. Introduction

Macrophages are highly adaptable innate immune cells that continuously adjust their phenotype and functions in response to signals from the surrounding tissue milieu. This plasticity enables them to participate in host defense, tissue homeostasis, and repair, but also to contribute to chronic inflammation and tumor progression when dysregulated. Although their behavior has lonsg been described within the binary M1/M2 paradigm, in vivo studies now indicate that macrophage activation spans a broad continuum of intermediate states rather than segregating into rigid categories, with cellular identity undergoing gradual, non-discrete transitions along this spectrum [1,2,3,4,5].

Historically, the M1/M2 framework was anchored in distinct arginine metabolic pathways—nitric oxide synthesis in M1-like cells versus arginase-driven ornithine and urea production in M2-like cells—a distinction that, while simplified, remains conceptually useful and is discussed in detail in Section 2 [6,7].

Beyond this dichotomy, macrophage behavior is deeply shaped by developmental origin and microenvironmental context. Tissue-resident populations derived from embryonic progenitors—including microglia, Kupffer cells, and alveolar macrophages—self-renew and acquire organ-specific transcriptional programs, while circulating monocytes continuously replenish tissues and contribute to inter-organ diversity. These ontogenetic determinants of macrophage identity are examined in detail in Section 2 [2,4,8,9].

Circulating monocytes are the primary precursors of tissue macrophages and are themselves phenotypically and functionally heterogeneous; their subset classification and functional profiles are described in detail in Section 2 [8,10,11,12].

In the following sections of the review, we will first summarize the main macrophage phenotypes and the molecular circuits that underpin their functional diversity, then examine how distinct tissue microenvironments and disease contexts sculpt macrophage behavior and finally discuss emerging therapeutic strategies that harness or redirect macrophage plasticity for clinical benefit [2,3,4].

## 2. Macrophage Phenotypic Diversity and Underlying Molecular Programs

Circulating monocytes are the primary precursors of tissue macrophage populations and are themselves phenotypically heterogeneous [8,13]. In humans, three subsets are distinguished by surface co-expression of CD14 and CD16, each with a distinct functional profile. Classical monocytes (CD14^++^CD16^−^) are the most abundant subset; they co-express the pro-inflammatory marker CD64 and the scavenger receptor CD163, reflecting a dual capacity for immune activation and tissue repair, and are rapidly mobilized to sites of inflammation. Intermediate monocytes (CD14^++^CD16^+^), once considered predominantly pro-inflammatory, are now recognized as primarily regulatory: they express high levels of antigen-presentation molecules (HLA-DR, CD80, CD86) together with repair-associated genes such as TGF-β, positioning them as modulators of the inflammatory response. Non-classical monocytes (CD14^+^CD16^++^) constitutively patrol the endothelial surface and display a mixed transcriptional profile combining inflammatory mediators with stress-response genes such as heme oxygenase-1 (HMOX1), enabling context-dependent pro- or anti-inflammatory functions [8,11,12,14]. Upon tissue recruitment, monocytes differentiate into monocyte-derived macrophages or monocyte-derived dendritic cells (mo-DC), complementing embryonically derived resident populations [14].

Macrophages exhibit a broad spectrum of activation states shaped by microbial stimuli, cytokines, metabolic cues and tissue-derived signals. Classical M1-like macrophages arise in response to Toll-like receptor (TLR) ligands such as lipopolysaccharide (LPS) together with IFN-γ or TNF-α. They upregulate MHC class II and costimulatory molecules (CD80, CD86) and secrete pro-inflammatory cytokines including IL-1β, IL-6, IL-12, IL-23 and TNF-α, as well as chemokines such as IL-8 and several C-C motif chemokine ligand (CCL) family members [2,3,15]. These cells reinforce Th1/Th17 immunity, enhance microbial clearance and contribute to antitumor responses. Their high expression of Fc and complement receptors facilitates recognition and uptake of opsonized pathogens, which are subsequently degraded within phagolysosomes through oxidative burst, nitric oxide production, antimicrobial peptides and acid-dependent hydrolases. Beyond pathogen killing, M1 macrophages remove apoptotic cells and necrotic debris, secrete matrix metalloproteinases (MMPs) and other proteases, and initiate angiogenesis through vascular endothelial growth factor (VEGF), basic fibroblast growth factor (bFGF) and TNF-α, supporting the early phases of tissue repair [2,3,15,16].

As inflammation resolves, macrophages progressively shift toward M2-like phenotypes that coordinate tissue repair and regeneration [2,3,4,17]. M2 macrophages express arginase-1 (ARG1), diverting arginine metabolism toward polyamine synthesis and matrix remodeling [16]. They secrete IL-10, IL-1RA, TGF-β and growth factors such as vascular endothelial growth factor (VEGF), epidermal growth factor (EGF) and placental growth factor (PlGF), promoting angiogenesis, extracellular matrix (ECM) deposition and wound healing. These cells contribute directly to vascular remodeling by releasing angiopoietins (Ang-1, Ang-2) and, in some contexts, acquiring endothelial-like features. M2 macrophages synthesize key ECM components—including collagens I, III and IV, fibronectin, tenascin-C and glycosaminoglycans—and regulate ECM turnover through MMPs (e.g., MMP-2, MMP-9) and their inhibitors (tissue inhibitors of metalloproteinases, TIMPs) [18]. Distinct M2 subsets refine these functions [2,3,4,16,17].

M2a, induced by IL-4/IL-13, promote debris clearance and matrix deposition through fibronectin, insulin-like growth factor (IGF), TGF-β and chemokines such as CCL17, CCL18 and CCL22 [2,3,4,16,19].

M2b, activated by immune complexes, produces a mixed inflammatory/regulatory cytokine profile [2,3,4,19].

M2c, driven by IL-10, TGF-β or glucocorticoids, mediate immunoregulation and late-stage matrix remodeling [2,3,4,19].

M2d, overlapping with tumor-associated macrophages (TAMs), arise via A2A receptor signaling and TLR engagement and secrete IL-10, VEGF and TNF-α, supporting angiogenesis and tumor progression [2,4,19,20,21,22].

M4, macrophages are induced by the platelet-derived chemokine CXCL4 (PF4), released from platelet α-granules upon activation, and represent a functionally distinct subtype that does not conform to the classical M1/M2 framework. Their phenotype is characterized by loss of CD163, upregulation of matrix metalloproteinase (MMP)-12, S100A8/A9, and cathepsins B/K, and reduced expression of scavenger receptors CD36 and SR-A. Notably, M4 cells resist canonical repolarization signals: they fail to upregulate M2 markers (CD206, CD200R) in response to IL-4/IL-13, and are unresponsive to IL-10 or glucocorticoid stimulation. These features translate into pro-atherogenic functions—impaired hemoglobin–haptoglobin clearance, reduced heme oxygenase-1 induction, and MMP-12-driven plaque destabilization—confirmed by the detection of CD68+CD163− macrophages in human coronary arteries whose prevalence correlates with plaque instability [23]. Additional conditioning by lipids and lipoproteins accumulating in the arterial interstitial fluid further skews tissue-resident macrophages toward dysfunctional, pro-atherogenic states [24].

The classical M1/M2 paradigm reflects divergent arginine metabolism: M1 macrophages convert arginine into nitric oxide and citrulline, supporting antimicrobial and antitumor functions, whereas M2 macrophages metabolize arginine into ornithine and urea, promoting cell growth and tissue repair. Although simplified, this framework remains useful when integrated with metabolic and transcriptomic profiling. M1 macrophages rely on glycolysis and express inducible nitric oxide synthase (iNOS), whereas M2 macrophages express ARG1 and markers such as CD163 and CD206, though hybrid phenotypes are common [2,3,4,16,25,26].

Macrophage identity is shaped by developmental origin and microenvironmental cues. Many tissues contain long-lived embryonically derived macrophages—such as Kupffer cells, microglia and alveolar macrophages—that self-renew and acquire organ-specific transcriptional programs. Circulating monocytes replenish tissues and differentiate into macrophages that complement resident populations. Tissue macrophages sense pathogens, damage and metabolic stress through PRRs such as TLRs and C-type lectins. Under homeostasis, naïve macrophages clear apoptotic cells and can polarize toward inflammatory or regulatory states depending on cytokines, growth factors, oxidized lipids and redox signals [2,4,9].

The lung exemplifies microenvironment-driven specialization. Alveolar macrophages, characterized by CD206 expression and transcriptional programs enriched for fatty acid binding protein 4 (FABP4) and peroxisome proliferator-activated receptor gamma (PPARG), maintain surfactant homeostasis, clear inhaled particles and apoptotic cells, and restrain excessive inflammation. Their regulatory profile includes high expressions of SERPING1, LIPA and CCL18. Single-cell and cytometry studies reveal multiple alveolar macrophage subsets defined by markers such as CD71, CD169 and CD274. Monocyte-derived macrophages recruited during inflammation can adopt similar transcriptional and functional features, highlighting the plasticity of the pulmonary macrophage niche [27].

**Figure 1:**
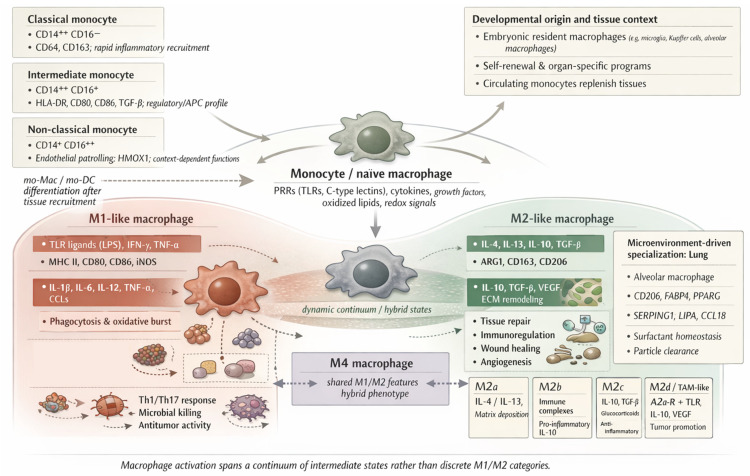
Simplified overview of macrophage polarization. Macrophages differentiate along a functional continuum in response to microenvironmental signals. Pro-inflammatory stimuli, such as LPS, IFN-γ, and TNF-α, promote an M1-like phenotype characterized by antigen presentation, inflammatory cytokine production, and antimicrobial or antitumor activity. Anti-inflammatory and repair-associated stimuli, including IL-4, IL-13, IL-10, and TGF-β, promote an M2-like phenotype characterized by tissue repair, extracellular matrix remodeling, and immunoregulation. Intermediate and hybrid states may coexist, reflecting the dynamic plasticity of macrophages.

The major macrophage phenotypes described in this section, together with their defining surface markers and key effector functions, are schematically represented in Figure 1.

Single-cell omics platforms, including scRNA-seq and CyTOF, have further demonstrated that macrophage activation exists along a continuum rather than discrete M1/M2 states [2,28]. TLR signaling can modulate M2 polarization, as TLR2 or TLR4 stimulation in the presence of IL-4 enhances expression of ARG1 and Ym1. IFN-γ activates STAT1 to drive M1 polarization, whereas IL-4/IL-13 activate STAT6 to induce M2 genes such as ARG1, CD206 and RELM-α. STAT1 and STAT6 exert reciprocal inhibition, shaping polarization outcomes. Nuclear receptors such as PPARγ and LXRs further promote M2-associated transcriptional programs [2,17,28,29,30].

MicroRNAs also fine-tune macrophage polarization. miR-155 enhances M1 responses by suppressing SOCS1 and SHIP1; miR-125b limits M1 activation by targeting IRF4; miR-146a provides negative feedback by downregulating IRAK1 and TRAF6. In contrast, miR-21, miR-124 and miR-223 promote M2 polarization by targeting PDCD4, C/EBPα and Pknox1, respectively [2,3]. Metabolic regulators further contribute: mTORC1 promotes M1 activation through HIF-1α and glycolysis, whereas AMP-activated protein kinase (AMPK) favors M2 polarization by inhibiting mTORC1 and enhancing oxidative metabolism. Epigenetic modifiers also participate, with HDAC3 supporting M1 traits and HDAC4/5 promoting M2 polarization [2,3,4,31,32]. Notably, therapeutic delivery of miR-21 or miR-124 enhances M2 polarization and accelerates inflammation resolution in murine models of sepsis and spinal cord injury [2,33].

Epigenetic regulation integrates environmental and metabolic signals. Chromatin modifications—including methylation, acetylation, ubiquitination, glycosylation, phosphorylation and lactylation—modulate gene expression without altering DNA sequence. Histone lactylation, identified in 2019, links lactate metabolism to transcriptional control. In hypoxic tumor microenvironments, lactate accumulation resulting from aerobic glycolysis (Warburg effect) promotes macrophage lactylation and drives M2-like polarization. M2 macrophages exhibit fragmented mitochondria, and impaired mitochondrial fusion increases lactate levels, enhancing histone lactylation and reinforcing anti-inflammatory programming [14,34].

Among tissue-resident macrophage populations, microglia represent the specialized myeloid sentinels of the central nervous system, exhibiting a distinct ontogeny from yolk-sac progenitors and CNS-specific polarization dynamics that are discussed in detail in Section 3.8 [15,33,35,36].

Single-cell technologies have substantially expanded the resolution at which macrophage diversity can be characterized. Using bulk transcriptomics, Xue et al. [37] profiled human macrophages exposed to 28 distinct activation conditions and identified 49 transcriptionally discrete subsets, illustrating the extensive plasticity of macrophage responses to environmental cues. Complementing this, Roussel et al. [28] employed CyTOF to map a continuous activation landscape, in which individual macrophages frequently displayed mixed inflammatory and reparative signatures rather than conforming to binary M1/M2 categories.

Epigenetic regulators further refine macrophage specialization. HDAC4 and HDAC5 facilitate STAT6-dependent transcription, thereby supporting the establishment of alternative-activation programs.

At the signaling level, IFN-γ activates STAT1 to drive M1-associated gene programs, whereas IL-4 or IL-13 receptor ligation activates JAKs and culminates in STAT6 phosphorylation; nuclear-translocated STAT6 then drives transcription of M2 signature genes including ARG1, CD206 and resistin-like molecule (RELM)-α. The reciprocal inhibition between STAT1 and STAT6 is a central switch governing polarization outcome, with STAT1 suppressing M2 differentiation and STAT6 counteracting M1 activation [2,3,4,6,17].

## 3. Dynamic Crosstalk Between Macrophages and the Pathological Tissue Microenvironment

### 3.1. Oncology

Many tumors show substantial infiltration by TAMs, which can represent a major fraction of the immune compartment. In several malignancies, higher TAM abundance associates with more aggressive features and worse clinical outcomes, though prognostic associations vary across tumor types and spatial niches [13,15]. TAM functional plasticity is a cornerstone of tumor microenvironment (TME) dynamics: macrophages in well-perfused regions tend toward M1-like activation, whereas persistent hypoxia and metabolic byproducts such as lactate stabilize hypoxia-inducible factor (HIF)-1α and sustain M2 polarization [2,38]. The IL-4/STAT6-dependent epigenetic programs discussed in Section 2 are not only preserved but amplified in the TME by tumor-derived mediators, consolidating a stable immunosuppressive TAM phenotype [2,3,14,17].

Tumor cells escape macrophage-mediated clearance through multiple innate immune checkpoints. The CD47/signal regulatory protein alpha (SIRPα), CD24/sialic acid-binding immunoglobulin-type lectin 10 (Siglec-10), and MHC-I/leukocyte immunoglobulin-like receptor B1 (LILRB1) axes collectively restrain TAM phagocytic function; c-Myc-driven overexpression of CD47 and programmed death-ligand 1 (PD-L1) further blunts T-cell immunity [39,40,41,42]. Costimulatory reprogramming via CD40–CD40L engagement can reverse this suppression, shifting TAMs toward a proinflammatory, tumoricidal phenotype. Regulatory T (Treg) cells, B cells, and tumor-secreted mediators (IL-10, VEGF, TGF-β, PGE_2_) further reinforce M2-like TAM states and impair antigen-presenting cell function [15,38,43,44].

**Figure 2:**
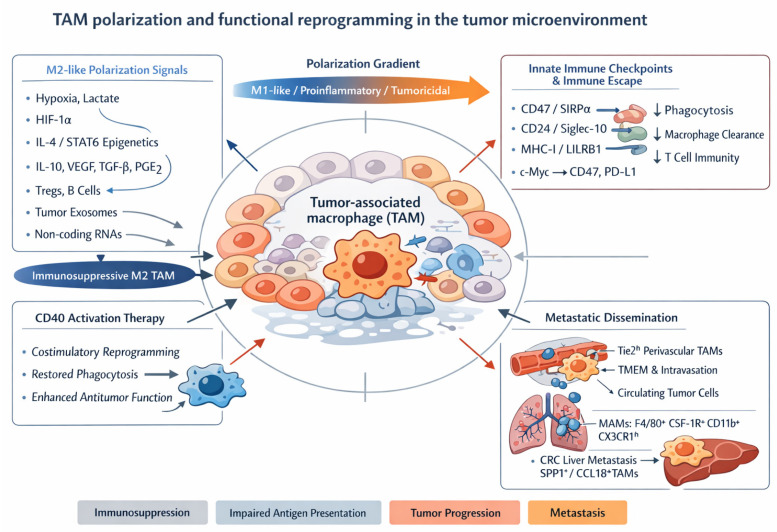
TAM polarization and functional reprogramming in the tumor microenvironment. Tumor-derived cues drive TAMs toward an M2-like, immunosuppressive phenotype. Metabolic factors (lactate, hypoxia/HIF-1α) and cytokines from tumor, Treg, and B cells (IL-4, IL-10, TGF-β, VEGF, PGE2) promote M2 polarization through IL-4/STAT6-dependent epigenetic programs. Tumor cells evade macrophage clearance via innate immune checkpoints (CD47/SIRPα, CD24/Siglec-10, MHC-I/LILRB1), while c-Myc-driven CD47/PD-L1 co-expression suppresses T-cell immunity and phagocytosis. Tumor-derived exosomes and ncRNAs further modulate TAM states. M2-skewed TAMs facilitate metastasis, with Tie2^hi TAMs promoting intravasation through TMEM structures and MAMs supporting metastatic outgrowth. Organ-specific niches imprint distinct transcriptional programs (e.g., SPP1+/CCL18+ TAMs in CRC liver metastases). CD40–CD40L engagement can redirect TAMs toward a proinflammatory, tumoricidal M1-like phenotype.

Macrophages also orchestrate metastatic dissemination: Tie2^hhhh^ perivascular TAMs facilitate intravasation via TMEM structures; metastasis-associated macrophages (MAMs, F4/80^+^/colony-stimulating factor (CSF)-1R^+^/CD11b^+^/CX3CR1^hhhh^) are recruited to the lung and are indispensable for metastatic outgrowth; organ-specific niches imprint distinct TAM transcriptional programs, as illustrated by SPP1^+^/CCL18^+^ TAMs enriched in CRC liver metastases [45,46]. The tumor-exosome axis and tumor-derived non-coding RNAs further fine-tune TAM polarization across solid tumor types, as detailed in the disease-specific subsections below [15,38,40,47]. These features are schematically illustrated in Figure 2.

#### 3.1.1. Breast and Ovarian Cancer

Within the perivascular niche, a distinct Tie2^high macrophage subset forms, together with Mena^high tumor cells and endothelial cells, the so-called TMEM (tumor microenvironment of metastasis) doorway, which serves as a transient portal for intravasation by locally increasing vascular permeability through CSF-1-driven, VEGF-A–dependent loosening of endothelial junctions [45,46].

The perivascular niche in the brain is especially critical because most cancer cells succumb to the robust neuroinflammatory response orchestrated by microglia and astrocytes; only those able to exploit this niche survive. In breast cancer brain metastases, large numbers of reactive astrocytes infiltrate metastatic foci and, upon activation, secrete chemokines such as IL-6 and TGF-β that function as oncogenic cues, enhancing tumor cell motility and invasiveness. In murine models of breast cancer brain metastasis, the long noncoding RNA Lnc-BM further promotes brain colonization by engaging JAK2–STAT3 signaling to induce ICAM1 and CCL2 expression, thereby facilitating breast cancer cell extravasation across the blood–brain barrier and recruiting macrophages to the nascent metastatic niche [46,48,49].

Functional studies manipulating macrophages in vivo in murine models underscore their importance for metastatic survival. Genetic perturbation of macrophage function in mice reduces the persistence of cancer cells in pulmonary capillaries and limits invasion into lung parenchyma, even in the presence of microthrombi, indicating a direct role for macrophages in supporting newly seeded cells. Mechanistically, recruited macrophages can activate phosphoinositide 3-kinase (PI3K)–protein kinase B (AKT) signaling in recently disseminated breast cancer cells via engagement of vascular cell adhesion molecule-1 (VCAM-1) through α4 integrins, thereby protecting tumor cells from proapoptotic cytokines such as TRAIL. In parallel, macrophage-derived chemokines and cytokines furnish additional survival cues that allow a subset of circulating tumor cells to withstand the hostile conditions of the metastatic microvasculature [46].

In breast cancer, CCL2 produced by tumor and stromal cells is a dominant chemokine that recruits CCR2^+^ inflammatory monocytes and differentiating macrophages to emerging metastatic sites and triggers a secondary chemokine cascade, including CCL3–CCR1 signaling, that stabilizes MAMs and drives lung micrometastasis [45,46,50]. Additional myeloid checkpoints also shape bone colonization: in patients with breast cancer bone lesions, CD137 expression on myeloid cells within bone promotes recruitment of monocytes/macrophages and their differentiation into osteoclasts, thereby fostering osteolytic metastases; targeting CD137 with liposomal anti-CD137 antibodies significantly reduces skeletal metastasis in vivo in murine models, emphasizing the protumoral role of CD137^+^ macrophages in this setting [51].

In ovarian cancer, TAMs are abundant—often constituting more than half of the cellular content of the tumor microenvironment, including ascitic fluid from patients with peritoneal spread—and actively participate in intraperitoneal dissemination. TAMs facilitate the formation of multicellular spheroids and induce P-selectin expression on mesothelial cells, thereby enhancing adhesion of tumor aggregates to the peritoneal surface. The omentum contains specialized immune aggregates known as milky spots, rich in macrophages, T and B cells, and vasculature; these structures—described in both murine models and human omental specimens—serve as privileged docking sites where tumor cells preferentially lodge and form secondary lesions, with resident macrophages secreting chemokines that actively support colonization and growth [46].

Beyond direct cytokine and chemokine signaling, tumor-derived extracellular vesicles represent an additional mechanism by which breast and ovarian cancer cells reprogram TAMs toward immunosuppressive phenotypes. In breast cancer, tumor exosomes shuttle miR-138-5p into macrophages, activating the miR-138-5p/KDM6B pathway and reinforcing M2-like programs that promote proliferation, angiogenesis, and metastasis [52]. Similarly, tumor cell-derived exosomal miR-191-5p activates M2-subtype macrophages through SOCS3 suppression, further promoting breast cancer progression [53]. In ovarian cancer, hypoxic tumor-derived exosomes deliver miR-940 to TAMs, driving acquisition of a tumor-supportive M2 phenotype that facilitates peritoneal dissemination [15,47].

#### 3.1.2. Lung Cancer

Lung cancer, and non-small cell lung cancer (NSCLC) in particular, develops within a complex immune microenvironment shaped by the unique cellular composition of the respiratory tract. Tumor-infiltrating macrophages interact with multiple immunoregulatory populations—including FOXP3^+^ regulatory T cells and myeloid-derived suppressor cells—to establish a suppressive network that restrains CD8^+^ cytotoxic T cells and upregulates checkpoint receptors such as PD-1 and lymphocyte activation gene-3 (LAG-3), contributing to T-cell exhaustion and impaired antitumor immunity [15,54]. Within this context, alveolar macrophages occupy a central and distinctive role.

Within the distal lung, alveolar macrophages (AMs) represent a highly specialized macrophage subset that is ontogenetically and functionally distinct from monocyte-derived macrophages. AMs originate predominantly from embryonic yolk-sac precursors and are maintained locally through self-renewal, largely independent of continuous input from circulating monocytes or bone marrow hematopoietic stem cells, enabling them to provide long-term surveillance within the alveolar space. Given their central role in preserving alveolar immune equilibrium, the phenotype of AMs in bronchoalveolar lavage samples is considered a sensitive indicator of the local microenvironment in patients with NSCLC, and flow cytometry remains the standard approach for detailed immunophenotypic analysis of lavage-derived leukocytes [55,56].

Multiplex immunofluorescence studies in NSCLC have shown that the vast majority of CD68^+^ AMs express the M2-associated scavenger receptor CD163, whereas a subset coexpresses the M1-associated costimulatory molecule CD86, consistent with a mixed but M2-biased polarization state. Notably, PD-L1 (CD274) staining in these cells reveals both membranous and nuclear patterns, suggesting complex regulatory roles beyond classical surface checkpoint engagement. Complementary Western blot analyses of additional polarization markers indicate relatively low expression of M1-linked molecules such as CD16 and inducible nitric oxide synthase, contrasted with higher levels of the M2 markers CD206 and arginase, collectively supporting the view that AMs in the NSCLC microenvironment are skewed toward an alternatively activated, immunosuppressive phenotype [55].

#### 3.1.3. Pancreatic Cancer

Pancreatic ductal adenocarcinoma (PDAC) arises in a characteristically fibrotic milieu, where an exuberant desmoplastic reaction generates a compact stromal scaffold that surrounds and interdigitates with neoplastic glands. This stroma is rich in activated fibroblasts and extracellular matrix components, which create a physical and biochemical barrier that impedes immune cell trafficking and diminishes drug penetration. Within this environment, TAMs are numerically prominent and, in established PDAC, are largely skewed toward an immunosuppressive, tumor-promoting M2-like state, typically marked by expression of CD163 and CD204, whereas proinflammatory, HLA-DR-high M1 macrophages are more characteristic of benign inflammatory pancreatic lesions. Clinically, an M2-dominated macrophage infiltrate associates with larger primary tumors, early hepatic relapse, local recurrence, and shortened overall survival [26,38,57].

Multiple cytokine circuits contribute to TAM-mediated immunosuppression in PDAC. TAM-derived TNF-α suppresses IL-33 production by PDAC cells, adding a layer of cytokine cross-regulation to the local niche. High intratumoral levels of CCL5 promote accumulation of CCR5-expressing macrophages and lymphocytes, and are implicated in tumor growth, therapeutic resistance, expansion of cancer stem-like populations, invasion, angiogenesis, and macrophage polarization toward an immunosuppressive M2 phenotype [45,57,58].

Functionally, M2-polarized TAMs in PDAC shape an environment that suppresses antitumor immunity and supports malignant progression. Compared with macrophages in nonmalignant settings, PDAC-associated TAMs produce less TNF while exhibiting constitutive secretion of IL-6 and IL-1, consistent with a chronic, tumor-supportive inflammatory state. They release chemokines such as CCL2 and CCL7 and immunoregulatory mediators including TGF-β, IL-10, and prostaglandin E_2_, which collectively foster T-cell exhaustion, recruit additional suppressive leukocytes, and stabilize an immune-excluded niche. CCL2 also drives CCL3 production by metastasis associated macrophages (MAMs), promoting their retention in metastatic sites. TAM-derived IL-10 inhibits natural killer (NK)-cell expansion, whereas TGF-β impairs NK cytotoxicity through contact-dependent mechanisms, further weakening innate immune surveillance. At the same time, macrophage expression of costimulatory and checkpoint ligands such as CD80/CD86 and PD-L1/PD-L2 engages CTLA-4 and PD-1 on T cells, dampening their activation, proliferation, and effector function. TAMs can also skew CD4^+^ T-cell differentiation away from Th1 responses toward Th2 or Treg phenotypes, thereby blunting cytotoxic T-cell-mediated tumor clearance [26,38,57,59].

At the molecular level, PDAC cells and stromal elements provide a continuous stream of signals that stabilize and expand the M2 TAM compartment. CSF-1, abundantly produced by tumor cells, drives monocyte recruitment and supports macrophage survival, differentiation, and motility. In a feed-forward loop described in murine PDAC models, CSF-1-activated macrophages secrete EGF, enabling coordinated migration of macrophages and tumor cells toward blood vessels, where macrophage-derived VEGF-A facilitates vascular breach and intravasation. Additional metabolic cues also reinforce M2 polarization: lactate efflux from PDAC lesions favors alternative activation, and M2 macrophages themselves can sustain their lineage through IL-10 production, further entrenching an immunosuppressive circuit [26,38,45,57].

Macrophage involvement in PDAC extends to the earliest stages of disease: in precancerous pancreatic intraepithelial lesions, inflammatory M1-like macrophages are actively maintained by CXCL10–CXCR3 signaling, suggesting that the myeloid microenvironment is shaped well before invasive carcinoma is established [60].

Intercellular communication via extracellular vesicles (EVs) and exosomes adds yet another layer of complexity to established PDAC. M2-type macrophages release EVs enriched in microRNA-301a-3p under hypoxic conditions; this miRNA targets TGF-β receptor 3, thereby amplifying TGF-β signaling in recipient PDAC cells. Uptake of these EVs promotes the epithelial–mesenchymal transition, as evidenced by loss of E-cadherin and upregulation of genes involved in motility and angiogenesis, conferring a more invasive, lymphotropic and vasculotropic phenotype [61]. Tumor-derived exosomes (TEXs) likewise contribute to immune escape by inducing apoptosis of CD8^+^ T cells, suppressing NK-cell activity, and fostering the expansion of Treg cells and myeloid suppressor populations, thereby broadening the immunosuppressive network beyond macrophages [62].

**Figure 3:**
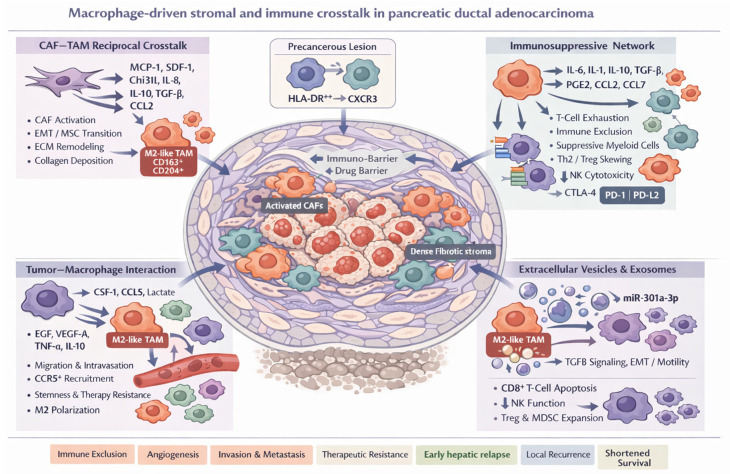
Macrophage-driven stromal and immune crosstalk in pancreatic ductal adenocarcinoma. Pancreatic ductal adenocarcinoma (PDAC) arises within a dense desmoplastic stroma enriched in CAFs, ECM components, and TAMs, which together limit immune-cell infiltration and drug delivery. In established PDAC, TAMs are largely M2-polarized (CD163, CD204), whereas HLA-DR^high M1-like macrophages prevail in early inflammatory or precancerous lesions driven by CXCL10–CXCR3 signaling. CAF-derived mediators (MCP-1, SDF-1, Chi3L1, IL-8, IL-10, TGF-β, CCL2) recruit monocytes and promote M2 polarization, while TAM-derived signals reinforce CAF activation, EMT, ECM remodeling, and fibrosis, establishing a self-sustaining stromal niche.

The interaction between stromal and malignant cells in PDAC is bidirectional and centrally involves cancer-associated fibroblasts (CAFs). CAFs shape the immunosuppressive microenvironment by promoting monocyte recruitment and their differentiation into M2-type TAMs through secretion of monocyte chemoattractant protein-1 (MCP-1), SDF-1, and Chi3L1, as well as IL-8, IL-10, TGF-β, and CCL2, which bias polarization toward pro-tumorigenic phenotypes. Flow cytometry analyses have shown that CAF-induced M2-type TAMs upregulate PD-1, a feature linked to impaired phagocytic activity and diminished T-cell infiltration [26,63,64,65]. In turn, M2-type macrophages enhance CAF activation and epithelial–mesenchymal transition (EMT) by releasing IL-6 and SDF-1 and can influence the trans-differentiation of mesenchymal stem cells (MSCs)—one of the progenitor populations giving rise to CAFs—further expanding the stromal compartment. The abnormal collagen-rich matrix generated by CAFs further recruits TAMs and reinforces M2 polarization, while TAM-mediated ECM remodeling progressively increases tissue stiffness, creating a self-sustaining pro-tumorigenic niche [38,57]. These features are schematically illustrated in Figure 3.

PDAC-associated TAMs suppress antitumor immunity through IL-6, IL-1, IL-10, TGF-β, PGE2, CCL2, and CCL7, driving T-cell exhaustion, immune exclusion, and recruitment of suppressive leukocytes. TAM-derived IL-10 limits NK-cell expansion, TGF-β reduces NK cytotoxicity, and macrophage CD80/CD86 and PD-L1/PD-L2 dampen T-cell activation via CTLA-4 and PD-1. Tumor-derived CSF-1 sustains macrophage recruitment and survival, while CSF-1-activated TAMs release EGF and VEGF-A to promote tumor-cell migration, vascular breach, and intravasation. Additional cues (CCL5, lactate, autocrine IL-10, TNF-α-mediated IL-33 suppression) stabilize the M2 phenotype.

Extracellular vesicles add a further regulatory layer: hypoxic M2 macrophages release EVs enriched in miR-301a-3p, enhancing TGF-β signaling and EMT in PDAC cells, whereas tumor-derived exosomes induce CD8^+ T-cell apoptosis, suppress NK activity, and expand Treg and myeloid suppressor populations. These stromal, immune, and metabolic interactions collectively drive immune evasion, invasion, angiogenesis, metastasis, therapeutic resistance, and poor clinical outcome in PDAC.

#### 3.1.4. Glioblastoma

Glioblastoma (GBM) harbors one of the most immunosuppressive tumor microenvironments among solid cancers, characterized by sparse T-cell infiltration, prevalent myeloid dominance, and frequent T-cell exhaustion. The myeloid compartment in GBM comprises two ontogenetically distinct populations: tissue-resident microglia, derived from yolk-sac progenitors, and monocyte-derived macrophages (MDMs) recruited from the circulation. These populations can be distinguished by flow cytometry and mass cytometry using differential expression of CD49d (ITGA4), FCGR2B, CLEC10A and CD209, which are enriched on MDMs but not on resident microglia [66].

High-dimensional profiling by mass cytometry and single-cell RNA sequencing has revealed that primary gliomas are enriched in tissue-resident CD49d^low microglia, while brain metastases from extracranial primaries such as breast, lung or melanoma harbor a larger fraction of CD49d^bright MDMs, reflecting a shift toward peripherally derived macrophages in the metastatic niche. Both microglia and MDMs undergo tumor-driven “education” that supports glioma progression, influencing tumor cell proliferation, invasion along white matter tracts and blood vessels, and responses to radiotherapy and systemic therapy [67,68].

Within glioblastoma (GBM), chemokine networks provide an important interface between glioma stem-like cells (GSCs) and TAMs. Mesenchymal GSCs secrete CXCL8, which sustains their own proliferation, survival and self-renewal through PI3K/AKT and NF-κB activation and simultaneously engages CXCR2 on TAMs to trigger a JAK2/STAT3 pathway that promotes an M2-like, immunosuppressive polarization state. Disruption of this CXCL8–CXCR2 axis in GSCs and TAMs reduces TAM M2 skewing, impairs GBM growth and prolongs survival in orthotopic models, highlighting a bidirectional loop that couples GSC maintenance to macrophage reprogramming [69]. Independent work has identified CD180 as another macrophage receptor that shapes glioma behavior: CD180 is upregulated in glioma-infiltrating macrophages, and CD180-overexpressing macrophages enhance glioma cell proliferation, migration, invasion and epithelial–mesenchymal transition while reinforcing immunosuppressive features in the tumor microenvironment, correlating with poorer prognosis [70].

At the transcriptomic level, GBM is a highly heterogeneous tumor classifiable into proneural, mesenchymal and classical molecular subtypes, each associated with distinct immune microenvironments. Macrophages derived from blood monocytes constitute the predominant immune infiltrate. Single-cell RNA-seq and cytometric analyses show that high-grade gliomas contain increased proportions of glioma-associated macrophages (GAMs), marked by genes such as Gpnmb and Spp1, together with intermediate monocyte/macrophage populations, while T-cell infiltration—particularly of CD8^+^ T cells—is reduced compared with lower-grade gliomas and non-neoplastic brain tissue. Across GAMs and related myeloid subsets in high-grade tumors, there is an enrichment of immunosuppressive mediators including Mif, Lgals3, Pkm, Axl, Id2 and Ccl4, whereas lower-grade lesions preferentially express chemokines that recruit effector T cells (Ccl5, Cxcl9, Cxcl10) and M1-associated genes such as Stat1, H2-Aa and IL-1β. Consistently, high-grade GBM specimens show increased CD74 and Id2 expression in CD45^hiCD11b^+^ myeloid cells, and CD74 frequently co-localizes with CD206^+^ M2-like macrophages, further supporting a skewing toward immunosuppressive myeloid phenotypes [66,67,68].

Mechanistic studies have delineated a STAT3-dependent CHI3L1/SPP1 positive feedback loop as a key driver of proneural-to-mesenchymal transition (PMT) and immune remodeling in GBM. CHI3L1 is highly expressed in mesenchymal GBM cells and promotes proliferation, clonogenic growth and migratory capacity, while osteopontin (SPP1), secreted by both tumor cells and macrophages, sustains NF-κB and STAT3 signaling and reinforces mesenchymal traits. Glioma cells overexpressing CHI3L1 can reprogram macrophages toward an M2-like phenotype characterized by increased CD206, CD163 and PD-L1, whereas STAT3 silencing reduces SPP1 expression and disrupts this feed-forward circuit. Metabolic cues further shape TAM states in GBM: intracellular lactate accumulation promotes H3K18 lactylation in glioma cells, CD4^+^ T cells and macrophages, increasing expression of CD39, CD73 and CCR8, promoting regulatory T-cell recruitment, and dampening Th17 responses—collectively reinforcing an immunosuppressive tumor milieu [71,72].

A particularly striking example of myeloid–tumor crosstalk is provided by “double-positive” TAMs that coexpress macrophage and glioma signatures. Single-cell and genomic analyses of human GBM specimens have identified a small subset of these cells, predominantly derived from bone marrow macrophages. Confocal imaging of patient-derived samples indicates that they arise through phagocytosis of glioma cells rather than cell fusion, as tumor DNA remains confined to the macrophage cytoplasm without nuclear mixing. Double-positive TAMs exhibit enhanced phagocytic features and high expression of M2-associated markers (MRC1, macrophage receptor with collagenous structure (MARCO)) as well as immune checkpoint molecules (PD-L1, PD-L2, CD276), while upregulating genes involved in T-cell suppression and regulatory cytokine signaling. Functionally, these cells dampen T-cell activation and promote immune escape, and similar double-positive populations have been reported in colorectal cancer, melanoma and ovarian cancer, suggesting a conserved mechanism by which phagocytic TAMs acquire tumor traits and adopt a strongly immunosuppressive, M2-like phenotype across distinct tumor types [18].

#### 3.1.5. Hematologic Malignancies (Leukemia and Lymphoma)

Leukemia and lymphoma cells actively remodel their myeloid niche through reciprocal signaling. Integrated single-cell RNA sequencing, flow cytometric, and immunohistochemical profiling of the innate immune compartment in the bone marrow of patients with acute myeloid leukemia (AML) has revealed a pronounced skewing toward an M2-like phenotype. Functionally, these AML-associated macrophages exhibit impaired phagocytic capacity, and intra-bone marrow coinjection experiments demonstrate that co-transplantation of M2-polarized macrophages with leukemic blasts markedly increases leukemogenic potential in vivo. Across patient samples, the macrophage compartment displays substantial interindividual heterogeneity; notably, individuals with a predominance of M2-polarized macrophages have particularly poor clinical outcomes, a pattern linked to reduced calreticulin (CALR) expression, a more stem-like transcriptional profile of leukemic blasts, and an intrinsic resistance of these blasts to macrophage-mediated engulfment. Direct physical contact with M2 macrophages further enhances leukemic cell homing and reshapes their metabolic state through the transfer of intact mitochondria from macrophages to blasts, providing an additional layer of metabolic support for leukemic stem-like cells. M2 macrophages drive leukemic transformation by imposing resistance to phagocytosis and improving mitochondrial metabolism [39].

To dissect transcriptional differences between AML-associated macrophages (AAMs) and macrophages from healthy donors, unsupervised clustering of scRNA-seq data (GSE116256) was performed, revealing a distinct gene expression signature enriched for M2/patrolling markers. Within this context, high expression of CD163 and CD206 correlates with adverse prognosis, in line with previous mRNA-based studies, and these markers retain prognostic value independent of standard clinical risk factors. Given the possibility that AAMs may arise directly from the malignant clone, leukemic blasts (CD45^dimHLA-DR^−^CD14^−^CD163^low) and AAMs (CD45^highHLA-DR^+^CD14^+^CD163^high) were sorted from AML bone marrow and subjected to sequencing, which demonstrated that canonical AML driver mutations present in blasts are also detectable within the corresponding AAM fraction, supporting a clonal relationship between leukemic cells and a subset of immunosuppressive macrophages [39,73].

Consistent findings across FLT3-ITD-positive cohorts showed that cord blood-derived macrophages harboring FLT3-ITD or DNMT3A knockdown exhibited significantly reduced phagocytic capacity compared with macrophages from healthy donors, indicating that acquisition of AML-associated mutations by macrophages can directly compromise their immune effector function [39]. Analysis of surface markers further revealed that expression levels of the canonical “don’t eat me” signals CD47 and CD24 inversely correlated with the extent of AML cell phagocytosis, whereas elevated display of the pro-phagocytic “eat me” signal calreticulin (CALR) associated with increased engulfment of leukemic blasts [74].

Beyond AML, accumulating data indicate that somatic mutations associated with clonal hematopoiesis of indeterminate potential (CHIP), such as TET2, ASXL1, and DNMT3A, when present in myeloid cells and macrophages, can drive a chronic proinflammatory state that favors expansion of mutant hematopoietic stem cell clones and perturbs bone remodeling within the marrow niche [75].

Additional experimental models have shown that genetic alterations confined to macrophages can modulate tumor susceptibility: macrophage-specific deletion of TP53 has been linked to increased incidence of intestinal neoplasia, and somatic TP53 mutations in leukemic cells can further skew the myeloid niche toward immunosuppression. In AML, leukemic blasts can, at least in part via their secretome, convert antitumor M1 macrophages into leukemia-supporting M2-like cells [39,75].

Macrophages also constitute a major stromal component in lymphoid malignancies, where they are referred to as lymphoma-associated macrophages. A high LAM burden has been associated with an aggressive clinical course and poorer outcomes in classical Hodgkin lymphoma, diffuse large B-cell lymphoma, follicular lymphoma, and angioimmunoblastic T-cell lymphoma [76,77].

At the signaling level, deregulation of the PI3K–AKT–mTOR axis is a hallmark of a sizeable subset of AML cases, often driven by lesions such as FLT3-ITD, which occur in about one-third of patients and are associated with dismal outcomes. Pharmacologic inhibition of PI3Kγ and PI3Kδ with the dual inhibitor IPI-145 reduces AML blast survival by blocking AKT-dependent prosurvival signaling and concurrently alters macrophage polarization, thereby targeting both leukemic cells and their myeloid microenvironment [78]. mTOR activity is likewise critical for macrophage fate: experimental data indicate that mTORC2 inhibition promotes a shift toward M2 polarization, whereas mTORC1 inhibition enhances M1 effector functions, suggesting that selective modulation of mTOR complexes may be leveraged to reprogram macrophage phenotypes in AML [79]. Clinically, reduced MOZ expression, together with elevated miR-223 levels, has been associated with monocytic AML subtypes, linking epigenetic and microRNA-mediated mechanisms to disordered macrophage development and function in leukemia [80].

M2-skewed lymphoma-associated macrophages (LAMs) frequently display immune checkpoint receptors such as PD-1 and PD-L1. This immunoregulatory phenotype is largely imposed by the surrounding cytokine milieu—dominated by IL-4, IL-13, IL-10, and M-CSF—generated by lymphoma cells and stromal elements, which drives alternative activation of macrophages. In primary cutaneous Diffuse Large B-Cell Lymphoma (DLBCL), leg type, as well as nodal DLBCL, the macrophage infiltrate is typically biased toward an M2 profile, and a predominance of M2 LAMs over M1 LAMs has been consistently linked to unfavorable clinical outcome. In line with this, Poles and colleagues [81] reported that Epstein–Barr virus (EBV)-positive DLBCL harbors a markedly increased M2 signature, reflected by a much higher CD163/CD68 ratio than in EBV-negative disease, underscoring the interplay between viral infection, macrophage skewing, and prognosis [75].

In T-cell acute lymphoblastic leukemia (T-ALL), contact with M2 macrophages markedly enhances leukemic proliferation through the release of soluble factors such as C5a, TNF, GRO family chemokines, and IL-6 [82]. In AML, Al-Matary et al. [83] demonstrated a pronounced enrichment of CD163^+^CD206^+^ M2-like TAMs in patient bone marrow compared with healthy controls, and Yang et al. [84] further associated increased CD163^+^ M2-like TAM counts with inferior survival, with splenic TAMs showing an even stronger M2 imprint than bone marrow TAMs [75].

In chronic lymphocytic leukemia (CLL), malignant B cells secrete nicotinamide phosphoribosyltransferase (NAMPT), which drives TAMs toward an M2-like CD163^hiCD206^hi phenotype via STAT3- and NF-κB-dependent pathways, resulting in macrophages that produce indoleamine 2,3-dioxygenase (IDO), IL-10, CCL18, IL-6, and IL-8 to sustain leukemic growth and inhibit effector lymphocytes. Additionally, apoptotic CLL cells release HMGB1, which promotes the differentiation of monocytes into nurse-like cells/TAMs, further consolidating a tumor-supportive milieu. In classical Hodgkin lymphoma (CHL), immunohistochemical analyses have shown that high densities of CD68^+^CD163^+^ TAMs associate with inferior overall survival, and TAMs account for a substantial fraction of PD-L1 expression in the tumor microenvironment, often colocalizing with PD-L1^+^ Hodgkin–Reed–Sternberg cells and forming close contacts with PD-1^+^ T cells. Complementary work by Vari et al. [85] demonstrated that PD-L1/PD-L2-expressing TAMs can dampen PD-1^hi NK-cell activity, an effect that is reversible upon PD-1 blockade; importantly, depletion of circulating monocytes from CHL patients before therapy enhanced activation of CD3^−^CD56^hiCD16^−^ NK cells, underscoring the central role of TAMs in immune escape and disease progression [75].

Angiogenesis and matrix remodeling constitute additional dimensions of macrophage-driven lymphoma biology. In follicular lymphoma, higher microvessel density and increased angiogenic sprouting correlate with elevated numbers of CD163^+^ TAMs and poorer prognosis. Several independent studies have also validated the negative prognostic impact of abundant TAMs or M2-polarized TAMs in systemic DLBCL and primary CNS DLBCL. Mechanistically, M2-TAMs in DLBCL can remodel the extracellular matrix through secretion of legumain, an asparaginyl endopeptidase that degrades fibronectin and collagen I, fostering tumor invasion and neovascularization [75,86].

**Figure 4:**
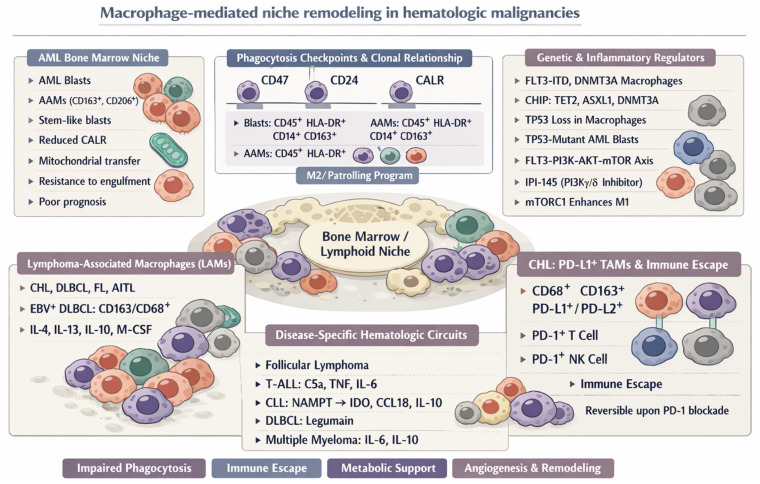
Macrophage-mediated niche remodeling in hematologic malignancies. Macrophages shape the leukemic and lymphoid microenvironment by regulating phagocytosis, immune escape, metabolic support, and tissue remodeling through disease-specific genetic and inflammatory programs.

In multiple myeloma, early observations by Zheng et al. [87] revealed dense infiltration of CD68^+^ macrophages in patient bone marrow compared with healthy individuals, with these macrophages promoting myeloma cell proliferation and shielding them from chemotherapy-induced apoptosis by preventing caspase-3 and poly (ADP-ribose) polymerase (PARP) activation and maintaining Bcl-xL expression. Subsequent work showed that macrophages, together with mesenchymal stromal cells, support myeloma growth and survival via secretion of IL-6 and IL-10, reinforcing the concept of macrophages as indispensable stromal partners in plasma cell malignancies [75]. These concepts are illustrated in Figure 4.

Unlike several previous reports, immunohistochemical evaluation of 118 DLBCL biopsies suggested that not all lymphomas conform to a strictly M2-dominated macrophage pattern: in this cohort, DLBCL tissues contained a higher proportion of M0 and M1 macrophages compared with control samples, indicative of a comparatively more proinflammatory macrophage milieu in at least a subset of DLBCL cases [88].

#### 3.1.6. Colorectal Cancer

The extracellular matrix (ECM) component elastin microfibril interfacer 2 (EMILIN-2), a member of the EDEN protein family, has emerged as a context-dependent tumor suppressor that shapes antitumor immunity by modulating macrophage polarization across different malignancies. In colorectal cancer (CRC) models, EMILIN-2 favors a proinflammatory M1 phenotype by engaging the TLR4/MyD88/NF-κB axis, whereas loss or downregulation of EMILIN-2 associates with an increased M2/M1 ratio and enhanced infiltration of alternatively activated, tumor-supportive macrophages [89].

TAMs also play a pivotal role in neovascularization, releasing a broad repertoire of proangiogenic mediators. In addition, TAM-derived thymidine phosphorylase and urokinase-type plasminogen activator enhance endothelial cell migration and contribute to ECM degradation, thereby indirectly facilitating angiogenic growth of the tumor vasculature [90].

At the level of liver metastasis, TAMs in CRC display a distinct transcriptional program characterized by SPP1, TREM2, glycoprotein non-metastatic melanoma protein B (GPNMB), and MARCO expression, closely resembling macrophage subsets found in pathological liver conditions such as lipid-associated macrophages in steatotic livers and scar-associated macrophages in fibrosis. Comparative analyses between macrophages in primary CRC tumors and liver metastases reveal a liver-specific enrichment of SPP1^+^ and CCL18^+^ TAMs, underscoring organ-specific imprinting of metastatic niches; among these markers, GPNMB has emerged as a key determinant of pro-tumor TAM identity and is strongly associated with poor prognosis [46,91]. Early-activated macrophages at metastatic sites express SERPINB2 and S100A8, whereas Kupffer cells and recruited monocyte-derived macrophages produce hepatocyte growth factor (HGF), which binds c-Met on tumor cells and promotes extravasation and engraftment within the liver parenchyma [46,92,93]. TAMs derived from ascites or solid tumors further contribute to tumor cell proliferation through HGF-1, IGF-1, and TGF-β secretion, and enhance invasiveness by activating NF-κB and c-Jun N-terminal kinase (JNK) pathways [2,46].

#### 3.1.7. Melanoma

In the melanoma TME, macrophages exhibit remarkable plasticity, as demonstrated in both human tumor specimens and murine models: they transition from pro-inflammatory, antitumor states to immunoregulatory phenotypes that promote tumor growth, angiogenesis, and immune suppression through mediators such as VEGF, TGF-β, and IL-10 [58,76,94].

Melanoma cells release chemokines and growth factors that recruit macrophages and bias them toward tumor-supportive phenotypes via TGF-β1, IL-10, and exosome-mediated signaling, as shown in patient-derived cell lines and in vivo murine melanoma models [47,76].

This plasticity is further reinforced by hypoxic microenvironmental cues and tumor-derived factors such as ADM and CD73, which shift arginine metabolism toward the M2 state and attenuate M1 responses [6,7,95].

### 3.2. Autoimmune and Rheumatic Diseases

Rheumatoid arthritis (RA) and osteoarthritis (OA) are among the most prevalent chronic joint disorders and, despite distinct etiopathogenic mechanisms, share inflammatory and immunological pathways that contribute to pain, dysfunction, and structural damage. RA is a systemic autoimmune disease affecting approximately 1% of the global population and is characterized by persistent synovial inflammation, immune cell infiltration, and progressive cartilage and bone destruction. OA, traditionally regarded as a degenerative condition, is increasingly recognized as a low-grade inflammatory disease involving the whole joint (cartilage, synovium, subchondral bone, and periarticular tissues), highlighting an important contribution of innate immune signaling and macrophage activity [96,97,98].

Macrophages are central regulators of joint homeostasis and disease progression in both RA and OA. Their remarkable plasticity allows them to adopt proinflammatory (M1-like) or reparative (M2-like) phenotypes depending on environmental cues. An imbalance favoring M1 over M2 macrophages contributes to disease progression by exacerbating inflammation and tissue destruction. This dynamic polarization is shaped by cytokines, chemokines, immune complexes, stromal interactions, and metabolic and epigenetic signals within the joint microenvironment [96,97,98].

The synovial membrane consists of an intimal layer composed primarily of macrophage-like and fibroblast-like synoviocytes and a subintimal layer containing stromal cells, blood vessels, and infiltrating immune cells. In RA, synovial fibroblasts play an active role in shaping macrophage recruitment and activation. They secrete chemokines such as CCL2 and CXCL8, which attract circulating monocytes into the synovial cavity, and produce proinflammatory cytokines including TNF-α, IL-1β, and IL-6, which promote M1 polarization. These interactions create a self-sustaining inflammatory niche that perpetuates macrophage activation and joint damage [96,97,99,100].

Stable RA remission is characterized by the enrichment of M2-like macrophage subsets, including M2a and M2c macrophages, in synovial tissues [96,98].

Adaptive immune cells further amplify macrophage-driven inflammation. Th1 and Th17 cells are key contributors to RA pathogenesis, promoting macrophage activation through cytokines and costimulatory interactions. Th17 cells attract monocytes via CCL2 and CCL20 and stimulate macrophages to produce IL-1β and TNF-α. A positive feedback loop arises in which macrophage-derived IL-23 enhances Th17 differentiation, while Th17-derived GM-CSF reinforces M1 polarization. B cells also contribute by producing rheumatoid factors and anti-citrullinated protein antibodies, which form immune complexes capable of activating macrophages to differentiate into M1 subtypes through Fc receptors, complement pathways, and TLR4, further amplifying inflammation [100,101,102,103]. Sokolove et al. [92] found that anti-citrullinated protein antibody (ACPA) produced by plasma cells in the synovial tissue can form immune complexes with citrullinated proteins; these immune complexes can then interact with macrophages through TLR4 and FcγRII to stimulate production of pro-inflammatory cytokines like TNF-α.

Macrophages are major effectors of structural joint damage. Their production of TNF-α, IL-1β, IL-6, and IL-23 stimulates synovial fibroblasts and chondrocytes to release matrix-degrading enzymes such as MMPs and aggrecanases (ADAMTS4, ADAMTS5), accelerating cartilage degradation. They also promote osteoclastogenesis by increasing receptor activator of NF-κB ligand (RANKL) expression on stromal cells and by differentiating into osteoclast precursors themselves [98,104].

A distinct macrophage population, known as arthritis-associated osteoclastogenic macrophages (AtoMs), has recently been identified within the synovial tissue of patients with RA. These CX3CR1^+^HLA-DRhighCD14^+^CD64^+^CD80^+^CD86^+^ cells localize near bone surfaces, interact closely with synovial fibroblasts, and possess a strong propensity to differentiate into osteoclasts, thereby directly contributing to bone erosion. Their phenotype and spatial distribution underscore their role as a critical interface between inflammation and structural damage [105].

Osteoclasts themselves originate from monocyte–macrophage lineage precursors, which undergo terminal differentiation in response to RANKL. In the inflammatory milieu of RA, macrophages amplify this process by releasing cytokines such as IL-6, TNF-α, and IL-1β. These mediators not only sustain synovial inflammation but also enhance RANKL expression on fibroblast-like synoviocytes, thereby promoting osteoclastogenesis and accelerating bone resorption [98,100,101].

Macrophage dysregulation is not limited to RA and OA but is a shared feature across multiple immune-mediated rheumatic and fibrotic diseases [3,96,106].

Systemic sclerosis (SSc) is a multisystem connective tissue disorder in which progressive cutaneous fibrosis is the most recognizable clinical feature, frequently accompanied by interstitial lung disease (SSc-interstitial lung disease (ILD)). A key initiating event is microvascular endothelial injury, which exposes neo-antigens and generates a milieu that promotes aberrant activation of innate immune cells. Cytokines such as IL-4, IL-13, and IL-10, together with vasoactive mediators including endothelin-1, contribute to the recruitment and functional reprogramming of circulating monocytes [106,107,108].

Peripheral blood monocytes from individuals with SSc display a distinct activation pattern. Classical CD14^+^ monocytes show increased expression of scavenger receptors such as CD163 and CD204, indicating a shift toward an alternatively activated, profibrotic phenotype compared with healthy controls [106,109,110]. Insights from next-generation RNA sequencing of early diffuse cutaneous SSc (dcSSc) skin biopsies have revealed an accumulation of CD68^+^ and CD163^+^ macrophages with transcriptional signatures strongly linked to fibrogenic pathways. Their presence in affected skin suggests that macrophage-derived signals play a central role in driving fibroblast activation and extracellular matrix deposition during the early phases of disease [107,111].

A second profibrotic macrophage population has been identified in the lungs of patients with SSc-ILD. These cells express SPP1 in response to IL-6 and TGF-β stimulation. Single-cell RNA sequencing has shown that SPP1^+^ macrophages from SSc-ILD lungs differ markedly from SPP1^−^ macrophages in healthy lungs, displaying distinct chromatin accessibility and transcription factor regulation. These findings indicate that the profibrotic lung macrophage phenotype is shaped by epigenetic mechanisms influenced by the local microenvironment [108,109].

In addition to tissue-resident macrophages, peripheral blood monocytes in SSc include subpopulations that co-express proinflammatory markers (CD80, CD86, TLR4) together with anti-inflammatory or scavenger receptors (CD163, CD204, CD206). These “hybrid” monocyte subsets correlate with clinical manifestations of the disease, particularly the presence and severity of SSc-ILD and the extent of skin fibrosis. Correspondingly, hybrid CD68^+^TLR4^+^CD163^+^CD204^+^CD206^+^ macrophages have been identified in lung biopsies and peripheral blood from patients with SSc-ILD, further supporting the idea that dysregulated macrophage activation contributes to both pulmonary and cutaneous fibrosis [109,112].

Macrophages also contribute to OA pathology through innate immune sensing mechanisms. They express pattern-recognition receptors (PRRs) capable of detecting pathogen-associated molecular patterns (PAMPs) and damage-associated molecular patterns (DAMPs). In OA, DAMPs arise from cartilage breakdown products—including aggrecan fragments, fibronectin fragments, and intracellular molecules released by necrotic chondrocytes. Engagement of PRRs by these endogenous ligands activates intracellular signaling cascades, most notably the NF-κB pathway, leading to the production of inflammatory cytokines and chemokines that perpetuate synovitis and drive further cartilage degradation [97,101].

In OA, macrophage activation is primarily driven by DAMPs released from degraded cartilage, including fibronectin fragments, aggrecan fragments, and intracellular proteins from necrotic cells. Engagement of pattern-recognition receptors activates NF-κB signaling and induces the release of inflammatory cytokines and chemokines that alter chondrocyte metabolism. This leads to excessive production of MMPs and ADAMTS proteases and accelerates extracellular matrix degradation. Recent findings indicate that M1 macrophages secrete R-spondin 2, which promotes terminal chondrocyte differentiation and increases MMP-13 expression, further exacerbating cartilage loss [97,101].

High-resolution transcriptomic profiling has revealed profound alterations in circulating monocyte subsets in systemic lupus erythematosus (SLE). Classical monocytes display a proinflammatory program dominated by type I interferon–responsive genes and enriched TNF/IL-1–related signatures, whereas non-classical monocytes undergo a distinct form of immune reprogramming and acquire a Th17-promoting phenotype, characterized by transcriptional modules that support IL-17-driven responses rather than a purely homeostatic surveillance profile. In skin and kidney lesions, macrophages infiltrating affected tissues present gene expression profiles that reflect intense crosstalk with stromal cells, particularly fibroblasts, and activation of TLR-dependent NF-κB pathways. These interactions contribute to the chronic inflammatory milieu typical of SLE target organs [112,113].

In lupus nephritis, macrophages with an M1-like phenotype express high levels of sphingosine-1 phosphate receptors (S1PRs). Engagement of these receptors by elevated circulating sphingosine-1 phosphate promotes activation of the NLRP3 inflammasome, leading to the transcription and release of multiple proinflammatory cytokines that amplify renal injury. Alongside these inflammatory populations, macrophages with profibrotic characteristics (particularly with M2a and M2c-like macrophages) also emerge during the later phases of renal involvement, where they participate in tissue remodeling and contribute to the incomplete resolution of inflammation [113,114].

Polymyalgia rheumatica (PMR) is characterized by a distinctive macrophage signature within synovial and periarticular tissues. Macrophages in the synovium and bursae simultaneously express inflammatory mediators and markers associated with tissue remodeling, reflecting a hybrid activation state that remains predominantly proinflammatory. This phenotype is shaped by cytokines such as IFN-γ, GM-CSF, and M-CSF, which collectively sustain macrophage activation and contribute to local inflammation [115].

Giant-cell arteritis (GCA) is characterized by a marked heterogeneity of monocyte-derived macrophages that populate different layers of the inflamed vessel wall. Once recruited into the arterial tissue, monocytes differentiate into specialized macrophage subsets whose distribution reflects the local microenvironment. Among these, CD206+MMP9+ macrophages accumulate in the adventitia during the earliest stages of disease, a process largely driven by T-cell-derived GM-CSF. As inflammation progresses, these cells migrate toward the media and the media–intima junction, where they contribute to extracellular matrix degradation and the development of intimal hyperplasia [110,116].

Classical CD14+CD16− monocytes appear to sustain the inflammatory response, whereas non-classical CD14+CD16++ monocytes that enter the vessel wall differentiate into CD68+CD16+CX3CR1+ macrophages, a population strongly implicated in tissue injury. Within the media and intima, macrophages upregulate iNOS and MMP9, promoting fragmentation of the elastic lamina and structural remodeling of the arterial wall. In the inner intima, gradients of M-CSF favor the emergence of folate receptor beta (FRβ)+ macrophages, which stimulate fibroblast proliferation and intimal thickening through PDGF-AA release and fibroblast activation [116,117].

Additional macrophage subsets arise as the inflammatory process evolves. GM-CSF supports the differentiation of CD206+MMP9+YKL-40+ macrophages, a population closely associated with tissue destruction and elastic lamina breakdown. In later disease stages, signals such as IFN-γ and TLR2 activation promote the fusion of macrophages into multinucleated giant cells. These CD68+CD163+MMP12+ giant cells produce mediators including M-CSF and PDGF, further amplifying intimal hyperplasia and vascular remodeling [110,116,118].

In psoriatic arthritis (PsA), synovial inflammation is strongly influenced by macrophage activity. CD163+ macrophages are particularly abundant within the synovial membrane and contribute to persistent inflammation by producing GM-CSF-associated mediators, including activin A, MMP12, and CCL17. These molecules support both synovitis and tissue remodeling, reinforcing the chronicity of the disease [117,119].

Although PsA pathogenesis is driven primarily by T-cell subsets—such as CD8+ T cells, Th1, Th17, Th9, and Th22 cells, the functional plasticity of synovial tissue macrophages (STMs) plays a crucial role, especially in early or undifferentiated forms of arthritis [119].

Across both early and established PsA, CD163+CD209+macrophages consistently show enhanced expression of activin A, GM-CSF and GM-CSF-responsive genes, including INHBA (encoding activin A), MMP12, TNF and CCL17. These factors collectively promote inflammatory amplification and extracellular matrix turnover [119,120].

**Figure 5:**
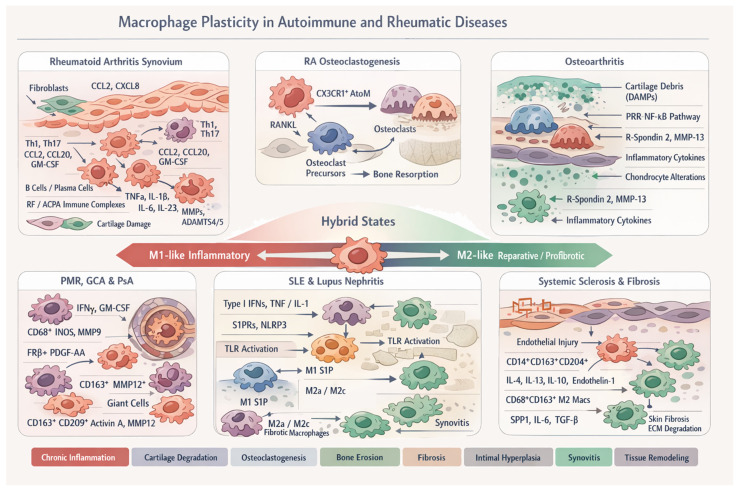
Macrophage plasticity in autoimmune and rheumatic diseases. Macrophages drive chronic inflammation, tissue remodeling, and structural damage across autoimmune and rheumatic diseases. In RA, synovial fibroblasts, Th1/Th17 cells, and immune complexes promote an M1-like state that fuels synovitis, cartilage breakdown, osteoclastogenesis, and bone erosion, while M2a/M2c-like phenotypes associate with remission. In OA, cartilage-derived DAMPs activate PRRs and NF-κB, sustaining synovitis and matrix degradation. In SSc, profibrotic and hybrid macrophage subsets (including SPP1+ lung macrophages linked to ILD) drive skin and lung fibrosis. In SLE/lupus nephritis, inflammatory monocyte/macrophage programs coexist with later profibrotic states involved in remodeling. Disease-specific macrophage subsets also promote vascular remodeling in GCA, persistent inflammation in PMR, and synovitis plus ECM turnover in PsA.

These concepts are illustrated in Figure 5.

### 3.3. Inflammatory Bowel Diseases

Inflammatory bowel diseases (IBD), including Crohn’s disease (CD) and ulcerative colitis (UC), develop in the context of a deeply altered immune landscape, involving coordinated disturbances across innate and adaptive immune compartments. A characteristic feature of the inflamed mucosa is the distorted equilibrium between pro-inflammatory and regulatory T-cell subsets, reflected in an overrepresentation of Th17 cells and a concomitant decline in Treg populations. This imbalance is reinforced by heightened release of IL-1 and TNF-α, coupled with inadequate production of IL-10, which collectively intensify the inflammatory drive. These processes progressively compromise the integrity of the mucus layer and epithelial barrier, creating a feedback loop that perpetuates immune activation and tissue injury [121,122,123].

Upon exposure to luminal antigens, the intestinal immune network (“immunome”) mounts a complex inflammatory response. Crohn’s disease typically exhibits a cytokine milieu driven by Th1 and Th17 pathways, with elevated secretion of IL-12, IFN-γ, and IL-17A, whereas ulcerative colitis is more closely aligned with a Th2-skewed immune profile characterized by increased IL-5 and IL-13. Additional innate lymphoid populations, including NK cells and ILC3s, contribute to the inflammatory environment by releasing IL-17 and IL-22, thereby reinforcing Th17-associated pathways. In UC, CD4+ Th2 cells and CD8+ T cells producing IL-5, IL-13, and IFN-γ predominate, while CD is marked by a strong Th1 signature with abundant TNF-α and IFN-γ release [121].

Among innate immune cells, macrophages represent a central hub in the initiation and perpetuation of intestinal inflammation. Both experimental and clinical evidence indicate a marked skewing of intestinal macrophages toward an M1-oriented phenotype, with a relative scarcity of M2 cells. M1 macrophages produce high levels of IL-12 and other pro-inflammatory mediators, and their activation is further shaped by genetic factors such as NOD2 variants and elevated immunity-related GTPase M (IRGM) expression, which interfere with inflammasome stability by promoting NLRP3 turnover and ultimately weakening NLRP1/NLRP3-dependent signaling. This M1-dominant environment amplifies mucosal inflammation and sustains chronic tissue damage [121,124].

Macrophage polarization is dynamically regulated by several endogenous and exogenous molecules. Several bioactive compounds—including baicalin, IL-33, selected lactic acid-producing microbes, and plant-derived flavonoids—have been shown in vitro and in murine colitis models to shift macrophage differentiation toward an M2-like, tissue-repairing phenotype, partly through activation of Wnt signaling pathways. Additional regulatory mechanisms involve microRNA-mediated control: diosgenin induces miR-125a-5p, while TNF-α upregulates miR-24-3p; both microRNAs enhance M2-like polarization and contribute to the attenuation of intestinal inflammation in preclinical settings. These findings highlight the therapeutic potential of targeting macrophage plasticity to restore immune homeostasis [2,121].

Despite these counter-regulatory pathways, macrophages infiltrating the intestinal mucosa during active disease frequently display heightened CD14 expression, robust NF-κB activation, and secretion of pro-inflammatory mediators that intensify mucosal damage and contribute to the pathogenesis of Crohn’s disease. Macrophage behavior is further shaped by intracellular signaling cascades: S1PR2 engagement activates the RhoA/ROCK1 axis to reinforce M1 programming, whereas yes-associated protein (YAP) signaling counteracts M2 differentiation and enhances IL-6 production [125].

One of the most clinically relevant consequences of macrophage-driven inflammation in Crohn’s disease is intestinal fibrosis. Fibrotic progression is strongly linked to colonization by adherent-invasive Escherichia coli (AIEC), a pathotype that exploits macrophages as its primary intracellular reservoir and drives chronic inflammation and aberrant tissue remodeling. Persistent AIEC survival within macrophages (demonstrated in both murine infection models and human intestinal macrophage cultures) promotes dysregulated wound healing and excessive extracellular matrix deposition, positioning macrophages as key orchestrators of CD-associated fibrogenesis [122,123,126].

### 3.4. Macrophage Plasticity in Non-Neoplastic Lung Diseases

Macrophages represent a major immune cell population within the respiratory tract and play essential roles in the initiation, amplification, and resolution of inflammation in asthma and other environmentally driven pulmonary disorders. Among the regulatory pathways influencing macrophage behavior, ATP6V0D2 has emerged as a key modulator. This lysosomal subunit, highly expressed in macrophages, functions as an inducible negative regulator of asthma severity by promoting the lysosomal degradation of Pu.1, a transcription factor involved in macrophage activation. Through this mechanism, ATP6V0D2 contributes to the dynamic control of macrophage polarization [127].

Macrophage-derived extracellular vesicles (EVs) can transport cytokines, lipids, microRNAs, and other bioactive molecules, thereby influencing inflammatory and fibrotic pathways across the lung microenvironment [128,129,130].

In severe asthma in children, chronic obstructive pulmonary disease, cystic fibrosis, and idiopathic pulmonary fibrosis, macrophage function is consistently impaired. These defects include reduced phagocytic capacity, inefficient efferocytosis, and increased susceptibility to apoptosis, all of which contribute to persistent inflammation and progressive tissue damage [128].

Following allergen exposure, macrophages with an M1-like phenotype release pro-inflammatory cytokines and chemokines that support pathogen clearance and facilitate the recruitment of T and B lymphocytes. Elevated levels of M1-associated markers have been reported in non-allergic asthma and correlate with more severe disease manifestations. Conversely, M2-like macrophages—typically associated with type 2 immune responses—are considered the predominant macrophage subset in allergic asthma and show a stronger association with disease activity [131].

In fibrotic lung diseases, macrophage phenotypes undergo additional pathological shifts. Alveolar macrophages isolated from patients with idiopathic pulmonary fibrosis (IPF) display markedly increased expression of profibrotic genes, including apolipoprotein E (APOE) and MMP12, reflecting their contribution to extracellular matrix remodeling. These cells also play a central role in efferocytosis, the clearance of apoptotic cells, a process mediated by several specialized receptors. Mer tyrosine kinase (MerTK) is one of the principal efferocytic receptors expressed by alveolar macrophages; upon recognition of apoptotic cells, MerTK activates phosphatidylinositol 3-kinase (PI3K) and Rac1, driving cytoskeletal rearrangements required for engulfment. TIM-4 further supports efferocytosis by binding phosphatidylserine exposed on apoptotic cell membranes [132,133,134].

In parallel, alveolar macrophages express signal regulatory protein-α (SIRPα), which interacts with surfactant proteins or CD47 on viable cells. Engagement of SIRPα activates Rho-associated kinase (ROCK) and the phosphatase and tensin homolog (PTEN), both of which antagonize PI3K signaling, thereby inhibiting Rac1 activation and suppressing efferocytosis. Dysregulation of this inhibitory axis can impair apoptotic cell clearance and contribute to chronic inflammation [135,136].

Evidence from IPF and other fibrotic lung diseases indicates that sustained M2 polarization is detrimental, as M2 macrophages secrete a broad array of profibrotic mediators. These include type 2 cytokines (IL-4, IL-13), chemokines (CCL2, CCL24), arginase-1, matrix metalloproteinases, and growth factors such as TGF-β, platelet-derived growth factor, and connective tissue growth factor. Collectively, these molecules promote fibroblast activation, extracellular matrix deposition, and progressive tissue remodeling [106,108,132,137].

In pulmonary fibrosis, enhanced glycolytic flux leads to intracellular lactate accumulation, which in turn promotes the expression of profibrotic mediators in naïve alveolar macrophages. This effect is mediated through histone lactylation, an epigenetic modification recently identified as a key regulator of macrophage gene expression. Histone lactylation has now been implicated in several fibrotic conditions, including pulmonary fibrosis and liver injury, suggesting that lactate-driven epigenetic remodeling may represent a unifying mechanism linking metabolic stress to macrophage-mediated tissue remodeling [14,136].

Macrophage dysfunction is also implicated in chronic lung allograft dysfunction (CLAD), a major cause of morbidity and mortality after lung transplantation, affecting up to half of recipients within five years. CLAD encompasses bronchiolitis obliterans syndrome (BOS), restrictive allograft syndrome (RAS), and mixed phenotypes. Immune profiling studies have shown that CLAD is characterized by a robust alloimmune response involving increased T cells—including CD8+ T cells—plasma cells, γδ T cells, eosinophils, and monocytes/macrophages, with a predominance of intermediate M2-like macrophages [134,138,139].

### 3.5. Macrophage Activation Syndrome (MAS) and Hemophagocytic Syndromes

Macrophage activation syndrome (MAS) represents a fulminant form of systemic hyperinflammation characterized by uncontrolled expansion and activation of tissue macrophages and cytotoxic lymphocytes, ultimately culminating in a cytokine storm. A defining pathological feature of MAS is the presence of mature macrophages or histiocytes exhibiting hemophagocytic activity. Similar immunopathological mechanisms are observed in hemophagocytic lymphohistiocytosis (HLH), in which activated macrophages display markedly increased expression of surface markers such as CD163, accompanied by substantial elevations in circulating soluble CD163 (sCD163). High CD163 expression—both in tissue macrophages and in serum—has been consistently associated with MAS and HLH occurring in the context of systemic juvenile idiopathic arthritis (sJIA), underscoring its relevance as a biomarker of macrophage hyperactivation [12,140,141].

### 3.6. Macrophage Polarization in Metabolic, Endocrine, and Cardiovascular Diseases

In acute myocardial infarction (MI), monocyte-derived macrophages (human and murine) undergo rapid activation accompanied by profound metabolic remodeling [20,142,143].

Elevated local lactate concentrations contribute to epigenetic reprogramming through histone lactylation, including modification of specific residues such as H3K18la, thereby enhancing the transcription of genes involved in monocyte-derived repair responses, such as Lrg1, Vegf-a and IL-10. IL-1β-dependent recruitment of GCN5 to target chromatin regions further supports this reparative transcriptional program. Conversely, experimental inhibition of histone lactylation in murine models is associated with increased systemic inflammatory cytokine levels, augmented post-MI cardiac inflammatory cell infiltration, impaired angiogenesis, adverse ventricular remodeling and deterioration of cardiac function, indicating that the lactate–lactylation axis promotes a shift toward a more reparative macrophage phenotype. Phenotypically, this can be interpreted as a progression from M1-like macrophages, producing high levels of pro-inflammatory mediators, toward populations enriched in IL-10 and M2-like markers, which can be monitored by tailored flow cytometry panels in experimental models [14,20,136,142,144].

A similar conceptual framework can be applied to skeletal muscle. Following muscle injury, lactate functions not only as a metabolic by-product but also as a key regenerative signal. In vitro, increased lactate concentrations promote myogenic cell differentiation, whereas in vivo (murine models) they support activation of muscle regeneration programs. At the molecular level, exogenous lactate induces histone H3 lysine 9 lactylation (H3K9la), thereby upregulating neuraminidase 2 (Neu2), a positive regulator of myoblast differentiation. In ischemic muscle injury models, these changes help restore a macrophage polarization pattern that favors tissue perfusion and regeneration. From a flow cytometry perspective, the temporal dynamics of macrophage responses in skeletal muscle can be tracked as an early influx of M1-like cells that progressively gives way to M2-like reparative subsets, characterized using M1/M2 surface markers and, where needed, intracellular reporters linked to metabolic pathways [14,145].

In adipose tissue, which serves not only as an energy reservoir but also as an active endocrine organ, macrophage polarization is tightly intertwined with systemic metabolic regulation. Visceral obesity, strongly associated with insulin resistance and increased cardiovascular risk, is characterized (in both humans and rodent models) by a microenvironment enriched in pro-inflammatory cytokines and chemokines. This milieu promotes the recruitment of monocytes and their polarization toward M1-like macrophages, whose metabolism is dominated by glycolysis, resulting in the conversion of pyruvate to lactate rather than entry into the tricarboxylic acid cycle. In contrast, M2-like macrophages under more physiological conditions rely primarily on fatty acid β-oxidation and oxidative phosphorylation (OXPHOS) to generate ATP, supporting tissue repair and resolution of inflammation [6,144,146].

Multiple endocrine and molecular cues modulate this balance. For example, adipocyte-derived miR-34a can limit M2 polarization by repressing Klf4 expression in murine adipose tissue, thereby sustaining chronic inflammation in obesity. Adipose tissue also secretes a broad array of adipokines, immune mediators and inflammatory factors that influence both adipocytes and resident macrophages, contributing to the development of obesity-related metabolic and inflammatory diseases, including inflammatory arthritis. Thyroid-stimulating hormone (TSH) signals through TSH receptors expressed in both adipocytes and macrophages, while mitochondrial components such as Cyclophilin D (CypD) participate in regulating mitochondrial function and, consequently, macrophage polarization. In flow cytometry, these alterations manifest as an expansion of CD11b+CD14+ populations expressing M1-like markers (e.g., CD80/CD86) and a relative decrease in CD163+/CD206+ subsets and can be further characterized by integrating intracellular readouts of NF-κB and JNK activation [119,144,147,148].

The link between obesity, adipose tissue inflammation and metabolic dysfunction extends to type 2 diabetes mellitus (T2DM) and end-organ complications such as diabetic kidney disease (DKD). In DKD, a persistent predominance of M1-like macrophages fuels chronic inflammation and fibrotic remodeling within the kidney. Autophagy has emerged as an important regulatory mechanism in macrophage polarization: high glucose levels suppress STAT3-dependent autophagy and drive macrophages (in vitro and in murine models of DKD) toward an M1 phenotype, while reducing the expression of M2-associated genes such as Arg-1 that are typically induced by IL-10 via STAT3 activation. Experimentally, flow cytometry can combine surface M1/M2 markers with intracellular autophagy indicators (e.g., LC3, p62) and phospho-STAT3 to directly link phenotypic profiles with signaling pathway status in renal or circulating macrophages [114,144,149].

Classical endocrine axes further shape the crosstalk between metabolism and innate immunity. In subclinical hypothyroidism, elevated TSH levels correlate with insulin resistance, and macrophages have been identified as one of the cell types with the highest TSH receptor (TSHR) expression outside thyroid follicular epithelium. In murine models with myeloid-specific deletion of Tshr (TshrMKO), exposure to a high-fat diet leads to less pronounced weight gain and a more favorable insulin sensitivity profile compared with control animals, accompanied by reduced infiltration of M1-like macrophages in the liver, adipose tissue and skeletal muscle. Co-culture experiments indicate that Tshr-deficient macrophages dampen gluconeogenesis in hepatocytes and enhance glucose uptake in adipocytes and myocytes by improving insulin signaling. Mechanistically, elevated TSH promotes M1 polarization by increasing the secretion of IL-1α, IL-1β and IL-6, which in insulin target tissues induce EGR1 and its downstream targets LCN2 and SOCS3, thereby exacerbating insulin resistance; blockade of IL-1 and IL-6 pathways mitigates these effects. In flow cytometric terms, this scenario translates into an expansion of CD11b+ pro-inflammatory macrophages expressing M1-like markers and a relative reduction in CD163+/CD206+ populations, with the option to simultaneously quantify intracellular IL-1β and IL-6 as functional correlates [148].

An illustrative example of the integration between immune signaling, lipid metabolism and endocrine function is provided by adrenal gland macrophages. These cells contribute to the regulation of glucocorticoid production by the adrenal cortex in response to stress. Expression of the receptor Triggering receptor expressed on myeloid cells 2 (Trem2) defines a population of lipid-associated macrophages with high phagocytic capacity and predominantly anti-inflammatory properties. Trem2 signaling requires adaptor proteins DAP10/DAP12 and activates downstream Syk–PI3K–ERK–Vav pathways, supporting TGF-β production by adrenal macrophages and thereby limiting excessive glucocorticoid synthesis. Conditional deletion of Trem2 in murine macrophages increases macrophage cell death, abrogates TGF-β production and leads to elevated serum glucocorticoid levels, along with upregulation of steroidogenic proteins in adrenal cortical cells; similar effects are reproduced by pharmacological blockade of TGF-β signaling. By flow cytometry, adrenal macrophages can be characterized by combining canonical myeloid markers with Trem2, while intracellular lipid accumulation is quantified using neutral lipid dyes such as Bodipy, which reveal higher lipid content in adrenal macrophages from atherosclerotic mice compared with non-atherosclerotic controls [150].

Macrophage involvement in autoimmune and fibro-inflammatory diseases is exemplified by thyroid-associated ophthalmopathy (TAO). Single-cell RNA sequencing analyses have shown that orbital tissues from TAO patients harbor distinct macrophage subsets, including CD163+ populations with high CCL18 expression that exhibit signatures of ferroptosis activation. Integrated single-cell and bulk transcriptomic data indicate that CD163+ tissue-infiltrating macrophages participate in the regulation of ferroptosis in orbital fibroblasts during TAO progression. Co-culture systems of human CD163+ macrophages with human orbital fibroblasts, together with in vitro models of fibroblasts stimulated with TGF-β1, have demonstrated that the TGF-β1/SMAD2/3 axis is a key regulator of ferroptosis in orbital fibroblasts, and that targeting macrophage-related ferroptotic signaling pathways may offer novel therapeutic opportunities. Flow cytometry can be used to identify and quantify CD163+/CCL18+ macrophages in orbital samples and to associate this phenotype with intracellular markers of ferroptosis and TGF-β/Smad signaling pathway (SMAD) pathway activation, thereby linking a tissue-specific M2-like profile to pathogenic functions [151].

Atherosclerosis is a chronic inflammatory disease initiated by the subendothelial retention and oxidative modification of low-density lipoproteins (LDL). Circulating Ly6Chi monocytes are recruited to sites of endothelial activation via adhesion molecules (VCAM-1), ICAM-1) and chemokine gradients (CCL2/MCP-1, CX3CL1), transmigrate into the intima, and differentiate into macrophages under the influence of M-CSF and GM-CSF. Once residing in the arterial wall, macrophages take up modified lipoproteins—primarily oxidized LDL (oxLDL)—through pattern-recognition scavenger receptors including CD36, SR-A1, and lectin-like oxidized LDL receptor-1 (LOX-1), becoming cholesterol-engorged foam cells that constitute the hallmark of early fatty streak lesions [24,146,152].

Macrophage polarization is a central determinant of plaque fate. Pro-inflammatory M1-like macrophages, driven by IFN-γ, LPS and oxLDL itself, dominate in early and unstable lesions: they release TNF-α, IL-1β, IL-6, IL-12, reactive oxygen species, and matrix metalloproteinases (MMP-1, MMP-9, MMP-12), promoting foam-cell apoptosis, impaired efferocytosis, and expansion of the necrotic core. Chronic impairment of efferocytosis—the phagocytic clearance of apoptotic cells—is a pivotal driver of necrotic core enlargement and plaque vulnerability, since uncleared apoptotic foam cells undergo secondary necrosis and release pro-inflammatory damage-associated molecular patterns (DAMPs). In contrast, anti-inflammatory M2-like macrophages, polarized by IL-4, IL-13 and IL-10, promote cholesterol efflux, suppress foam-cell formation, enhance efferocytosis, and support fibrous cap stability, thereby exerting atheroprotective effects [24,146,152,153].

Beyond the classical M1/M2 dichotomy, single-cell transcriptomic profiling of human atherosclerotic plaques has revealed multiple functionally distinct macrophage subsets, including TREM2hi foamy macrophages enriched in lipid-processing genes (FABP5, LGALS3), resident-like macrophages, and inflammatory subsets expressing IL-1β and NLRP3 inflammasome components. TREM2hi macrophages exhibit dual roles: at early stages they promote oxLDL uptake and foam-cell survival, whereas at advanced stages they limit necrotic core formation and enhance efferocytosis, illustrating the context-dependency of macrophage function within the plaque microenvironment. Plaque macrophages also display metabolic plasticity: M1-like cells rely on glycolysis and HIF-1α-driven programs, while M2-like and TREM2hi cells preferentially use oxidative phosphorylation and fatty acid oxidation—metabolic profiles that directly shape their inflammatory output [154].

The M4 macrophage subtype, induced by the platelet-derived chemokine CXCL4 and characterized by loss of CD163, elevated MMP-12 and S100A8/A9, and resistance to standard M2 polarization signals, represents an additional pro-atherogenic population enriched in human coronary plaques, where its prevalence correlates with features of plaque instability [23]. Taken together, the polarization balance and metabolic state of macrophages within the plaque microenvironment—not simply their abundance—determine whether atherosclerotic lesions progress toward instability and rupture or stabilize and regress [23,144,146,152,153,155].

**Figure 6:**
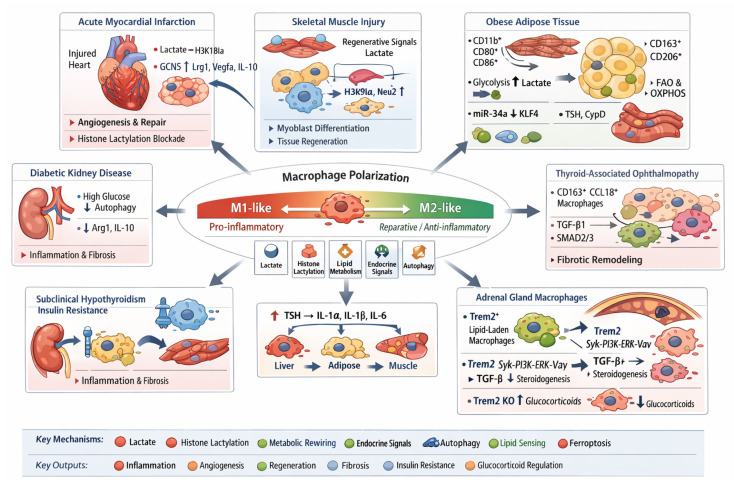
Metabolic and endocrine microenvironments shape macrophage polarization and functional plasticity across tissue contexts. Macrophage polarization in metabolic and endocrine diseases is driven by tissue-specific metabolic, epigenetic, and hormonal cues. Lactate promotes reparative histone lactylation programs in myocardial infarction and skeletal muscle injury; obesity shifts adipose macrophages toward a glycolytic M1-like state, whereas physiological conditions favor OXPHOS-dependent M2-like polarization. High glucose sustains fibrotic M1-like macrophages in diabetic kidney disease, elevated TSH promotes macrophage-derived IL-1 and IL-6 in subclinical hypothyroidism, Trem2+ adrenal macrophages regulate glucocorticoid production, and CD163+CCL18+ macrophages drive fibroblast activation in thyroid-associated ophthalmopathy. Flow cytometry can integrate M1/M2 markers with functional readouts of autophagy, inflammation, lipid sensing, and ferroptosis. Abbreviations: AMI, acute myocardial infarction; DKD, diabetic kidney disease; TAO, thyroid-associated ophthalmopathy; OXPHOS, oxidative phosphorylation; TSHR, thyroid-stimulating hormone receptor.

These concepts are illustrated in Figure 6.

### 3.7. Macrophage Plasticity in Infectious Diseases

Macrophages are central innate immune cells that rapidly counter pathogens through phagocytosis and antigen presentation. Their polarization during chronic infection illustrates the functional plasticity required to balance tissue homeostasis and immune regulation. Prolonged inflammatory stimulation triggers an early oxidative burst followed by oxidative stress and metabolic reprogramming, driving the shift from an M1- to an M2-oriented phenotype. This transition involves remodeling of the tricarboxylic acid (TCA) cycle and increased reliance on oxidative phosphorylation, fatty acid oxidation, and glutamine metabolism [6,144].

In chronic infections such as tuberculosis and human immunodeficiency virus (HIV), polarized macrophages release immunomodulatory mediators, including IL-10 and TGF-β, which contribute to lesion maturation and fibrosis while promoting an immunosuppressive environment that favors pathogen persistence [156,157].

In the field of infectious diseases, numerous pathogens remodel macrophage metabolism to reshape their functional programs, thereby promoting immune evasion and long-term persistence during chronic infection. As a result, macrophage metabolic reprogramming has emerged as a key focus in the study of chronic infectious diseases. In the early phases of persistent infection, macrophages typically adopt a pro-inflammatory M1-like phenotype to constrain pathogen replication. Over time, however, microbial products and pathogen-derived metabolites progressively rewire host metabolic pathways, driving a functional shift in macrophages. This reprogramming frequently culminates in a transition from an M1-dominated response to an anti-inflammatory M2-like state, which dampens antimicrobial activity and facilitates pathogen survival within the host [156,158].

Emerging data underscore that macrophage states are not shaped by pathogens alone but are profoundly modulated by epithelial–immune and stromal–immune crosstalk within affected tissues. In COVID-19, for example, disrupted interactions between infected airway epithelium, stromal compartments and the myeloid compartment favor the accumulation of hyperinflammatory blood monocytes and lung macrophages, which fuel cytokine production and drive alveolar damage and respiratory failure [159,160].

At the signaling level, STAT1 is a central driver of M1 polarization, and the JAK/STAT1 pathway is also implicated in IFN-γ-induced ferroptosis across several cell types. This axis represents an important component of the early innate response in infections such as Helicobacter pylori, where it contributes both to host defense and to immune escape mechanisms that underpin gastric carcinogenesis. Exosomal CagA derived from H. pylori not only skews macrophages toward an M1-like phenotype but also triggers ferroptotic cell death. In gastric biopsies from patients with CagA-positive H. pylori gastritis, elevated iNOS expression, together with reduced SLC7A11, highlights a pathogen-driven coupling of M1 polarization and ferroptosis in macrophages [161].

Iron handling provides an additional layer of complexity in pathogen–macrophage interactions. Legionella pneumophila, a facultative intracellular bacterium, secretes the polycarboxylate siderophore rhizoferrin, which supports bacterial growth under iron-restricted conditions and enhances survival in the murine lung. L. pneumophila enters the respiratory tract via inhalation of contaminated droplets and replicates within macrophages using an intracellular pathway that resembles its life cycle in amoebae. Genetic studies indicate that rhizoferrin biosynthesis (lbtA) and ferrous iron uptake via FeoB are partially redundant for iron acquisition. A double lbtA/feoB mutant shows profound growth impairment even under modest iron limitation, demonstrating that rhizoferrin-mediated ferric iron uptake and FeoB-dependent ferrous iron uptake are both critical for iron homeostasis. This mutant is also defective in biofilm formation, uncovering a role for rhizoferrin in extracellular survival. Importantly, the lbtA/feoB mutant displays markedly reduced replication in amoebae and in human U937 macrophage-like cells, revealing that rhizoferrin contributes to intracellular infection. Purified rhizoferrin can stimulate cytokine production by U937 cells, indicating that this siderophore not only supports bacterial nutrition but also modulates the inflammatory milieu; close homologs are found in Aquicella siphonis, another intracellular parasite of amoebae. During host defense, IFN-γ-activated macrophages reduce intracellular iron availability, thereby restricting Legionella growth [162].

Serosal cavities provide another example of how macrophage subsets contribute to infection control and tissue repair. Serous membranes line and protect internal organs and harbor diverse immune populations, among which macrophages represent a major component. In the pleural cavity, “small” serosal macrophages (MHCII+F4/80−) account for approximately 10% of resident macrophages, whereas “large” serosal macrophages (MHCII−F4/80+) constitute the remaining majority. Using flow cytometry and imaging, it has been shown in murine models that labeled pleural macrophages can migrate into the lung during the resolution phase of influenza A virus infection. Functional depletion of pleural macrophages prior to influenza infection results in greater weight loss, prolonged recovery, higher inflammatory cytokine levels in both pleural space and lung parenchyma, and increased neutrophil influx into the lungs. These findings suggest that pleural macrophages are recruited when restoration of homeostasis becomes critical, contributing to a pool of interstitial macrophages (including nerve- and airway-associated macrophages) that proliferate and expand following infection [163].

Macrophage metabolic reprogramming also shapes the outcome of helminth infections and related cardiovascular and metabolic sequelae. Chronic helminth infection has been epidemiologically associated with a reduced risk of coronary artery disease and a more favorable metabolic profile. Experimental studies suggest that helminth-induced type 2 cytokines, particularly IL-4 and IL-13, can drive macrophages toward a tissue-repairing, M2-like phenotype that supports resolution of inflammation within atherosclerotic lesions. In parallel, these cytokines help maintain adipose tissue metabolic homeostasis, and the combination of enhanced M2 polarization and preserved adipose function may act in concert to slow atherosclerosis progression [2,164].

Parasitic infections provide additional insights into macrophage plasticity. Schistosomiasis is characterized by egg-induced granulomatous inflammation and progressive hepatic fibrosis. Monocyte-derived macrophages exhibit considerable plasticity during both progression and regression of Schistosoma-induced liver disease. Mammalian STE20-like protein kinase 1 (MST1), a serine/threonine kinase, has been identified as a negative regulator of macrophage-driven inflammation. In infection with Schistosoma japonicum, macrophage-specific MST1 appears to limit both inflammatory responses and fibrotic remodeling. Mice with macrophage-targeted deletion of Mst1 develop more severe hepatic pathology with larger granulomas and increased fibrosis, accompanied by higher levels of pro-inflammatory cytokines (IL-1β, IL-6, IL-23, TNF-α and TGF-β). Mechanistically, activation of MST1 by soluble egg antigens enhances PPARγ-dependent CD36 expression, promoting phagocytosis and induction of fibrolytic genes such as Arg1 and matrix metalloproteinases. Loss of MST1, by contrast, suppresses fibrolytic gene expression while amplifying pro-inflammatory programs and impairing phagocytosis, thereby exacerbating liver fibrosis [165].

Macrophage migration inhibitory factor (MIF) is another key player at the intersection of inflammation, infection and cell death. It exerts its effects through several receptors—CD74, CXCR2, CXCR4 and CXCR7—and functions as a non-cognate ligand for CXC chemokine receptors, thereby influencing the trafficking of monocytes, macrophages, T cells and B cells [166]. In certain viral infections, such as HIV, MIF can help create a microenvironment that favors viral replication and immune evasion, promoting persistence of infection [157].

In dengue virus infection, serum MIF concentrations correlate with disease severity, and patients who succumb to dengue hemorrhagic fever exhibit higher MIF levels than survivors or patients with milder disease. MIF-deficient mice display lower viremia, reduced pro-inflammatory cytokine production and delayed lethality, supporting a pathogenic role for MIF in dengue [167].

Macrophage migration inhibitory factor (MIF) modulates macrophage polarization in a pathogen- and context-dependent manner. In HIV-infected monocyte-derived macrophages, exogenous MIF boosts pro-inflammatory cytokine release (IL-1, IL-6, IL-8, TNF-α, sICAM), amplifying M1-like responses that can paradoxically support viral persistence [157]. In contrast, MIF is protective in respiratory syncytial virus (RSV) [168], Toxoplasma gondii [169] and Aspergillus fumigatus [170] infections, where its absence or blockade increases pathogen burden and mortality, whereas in Candida albicans [171] and Plasmodium yoelii infection [172] its effects on inflammation and outcome are more nuanced and stage-dependent. Clinically, MIF levels are consistently elevated in sepsis, supporting its use as a biomarker and potential therapeutic target [30].

D-dopachrome tautomerase (D-DT/MIF-2) is a MIF homolog that also activates pro-inflammatory signaling and opposes glucocorticoid actions, but with distinct receptor usage and structural motifs. In sepsis and LPS-driven inflammation, both MIF and D-DT are upregulated, yet their impact on M1/M2 balance can be opposite in specific tissues (e.g., adipose tissue), indicating that the relative dominance of MIF vs. D-DT helps fine-tune macrophage activation states in infection and systemic inflammation [173].

Regarding fungal infections, the caspase recruitment domain-containing protein 9 (CARD9) is essential for directing monocyte-derived macrophages toward an effective antifungal program (as demonstrated in murine models of fungal infection) rather than a TREM2^hi state marked by elevated Trem2, Lgals3, and Apoe expression and reduced microbicidal capacity. In the absence of CARD9, macrophages still accumulate at the lesion site but predominantly adopt this TREM2^hi profile, characterized by restrained NF-κB activation, enhanced cAMP response element-binding protein (CREB)/C/EBPβ signaling, and impaired fungal clearance. CARD9 deficiency also alters the adaptive antifungal response by promoting the accumulation of Th1 cells with an exhaustion-like profile, which are less effective at sustaining protective immunity. This disrupted communication between macrophages and T cells further compromises fungal control, underscoring how innate macrophage programming can influence the strength and persistence of adaptive responses [174].

Finally, in highly inflammatory conditions such as bacterial pneumonia or acute respiratory distress syndrome, circulating CD14+ monocytes are recruited to the lung and differentiate into alveolar and interstitial macrophages. Receptors such as CD163 can act as binding partners for both Gram-positive and Gram-negative bacteria, further illustrating the dual role of macrophages as sensors and effectors in host defense. However, diminished antimicrobial activity and a preferential reliance on oxidative metabolism in M2-like macrophages—compared with the glucose-dependent, highly microbicidal machinery of M1 cells—can facilitate pathogen persistence. Understanding how infections, endogenous mediators and biomaterials collectively tune this M1/M2 and metabolic balance is therefore crucial for designing strategies that both control pathogens and preserve tissue integrity [12,30,144].

**Figure 7:**
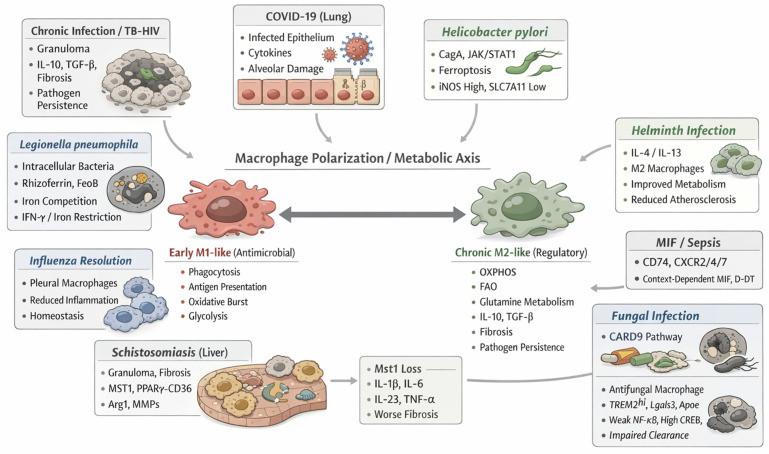
Macrophage plasticity and metabolic reprogramming in infectious diseases. In infectious diseases, macrophages undergo context-dependent metabolic reprogramming that governs host–pathogen outcomes. M1-like responses (glycolysis, oxidative burst, STAT1/JAK) shift toward M2-like states (OXPHOS, fatty acid oxidation (FAO), IL-10/TGF-β) during chronic infection, reducing microbicidal activity. Disease-specific mechanisms include: (i) JAK/STAT1-driven ferroptosis in H. pylori (iNOS ↑/SLC7A11 ↓); (ii) rhizoferrin/FeoB-mediated iron acquisition sustaining L. pneumophila replication; (iii) pleural macrophage migration limiting cytokine storm during influenza A resolution; (iv) MST1/PPARγ/CD36 promoting fibrolysis in S. japonicum; (v) MIF and D-DT fine-tuning M1/M2 balance in HIV, dengue, RSV, and sepsis; and (vi) CARD9-directed antifungal programming preventing TREM2hi immunosuppression. Epithelial–stromal–immune crosstalk, exemplified by coronavirus disease 2019 (COVID-19) hyperinflammatory macrophage accumulation, further shapes these transitions. Abbreviations: MIF, macrophage migration inhibitory factor; D-DT, D-dopachrome tautomerase; MST1, mammalian STE20-like kinase 1; CARD9, caspase recruitment domain-containing protein 9; FAO, fatty acid oxidation; OXPHOS, oxidative phosphorylation.

These concepts are illustrated in Figure 7.

### 3.8. Macrophage Plasticity in Non-Neoplastic Neurological Diseases

Neuroinflammation is a central pathogenic mechanism in a broad spectrum of central nervous system (CNS) disorders, including Alzheimer’s disease, Parkinson’s disease, Huntington’s disease, multiple sclerosis, traumatic brain and spinal cord injury, encephalitis and ischemic stroke. Both resident glia and peripheral immune cells contribute to this process. In ischemic strokes, for example, a rapid rise in circulating leukocytes accompanies tissue injury (observed in both human patients and murine models), with early neutrophil accumulation tightly correlating with infarct size and subsequent recruitment of monocytes and lymphocytes over days to weeks, while astrocytes undergo one of the earliest and most prominent activation responses following ischemic damage [34,36,175,176].

Transient depletion of resident microglia in the early phase after ischemic stroke in murine models profoundly reshapes the cerebral immune environment, facilitating the recruitment and expansion of a distinct population of monocyte-derived macrophages with a pro-reparative transcriptional and metabolic program. These repair-oriented macrophages contribute to white-matter preservation, angiogenic remodeling, and sustained neurological recovery. Their activity is partly governed by the transcription factor Mafb, which supports the acquisition of regenerative functions and promotes long-term tissue repair [175].

Microglia and CNS-infiltrating macrophages integrate PAMPs and DAMPs via pattern-recognition receptors such as TLRs and purinergic receptors, which drive their polarization along an M1/M2-like continuum. In the presence of IFN-γ and LPS, resting microglia are biased toward an M1-like phenotype that produces high levels of pro-inflammatory mediators (TNF-α, IL-1β, IL-6, CCL2, NO, reactive oxygen species (ROS)), disrupts blood–brain barrier integrity and amplifies neuroinflammation. Inflammasome activation is a key component of this response: the NLRP3 inflammasome, predominantly expressed in microglia, activates caspase-1 and promotes IL-1β and IL-18 release, worsening neurological dysfunction in ischemic stroke, intracerebral hemorrhage and substance use disorders, while NLRP1 inflammasomes engage neurons in the inflammatory cascade and can induce neuronal pyroptosis [177,178,179].

By contrast, M2-like microglia/macrophages exert largely neuroprotective functions. These cells can be induced by IL-4, IL-13, IL-10 and TGF-β through STAT3/STAT6, CB2R or TLR4-linked pathways, leading to increased production of anti-inflammatory cytokines and neurotrophic factors such as IGF-1, Arg1, FIZZ1, CD206, Ym1/Chi3l3 and TGF-β. In experimental models, promoting M2 skewing reduces pro-inflammatory cytokine secretion, attenuates demyelinating lesions in autoimmune encephalomyelitis and enhances hippocampal neurogenesis under chronic stress. Nevertheless, M2 cells often exhibit lower phagocytic activity than M1 cells, which can be detrimental for efficient clearance of debris and may limit optimal repair. In stroke and spinal cord injury, an early predominance of M2-like microglia/macrophages during the first days is typically followed by a delayed shift toward a more destructive M1-like phenotype around one week after injury, coinciding with persistent inflammation and secondary damage [180,181].

NF-κB occupies a central position in this network, controlling transcription of chemokines, cytokines, adhesion molecules and other inflammatory mediators in neurons, glia and vascular cells. NF-κB signaling can have dual roles, contributing to neuronal survival and repair in some contexts while driving chronic inflammation and neurodegeneration in others, depending on cell type, activation strength and temporal dynamics [182,183].

In neurodegenerative diseases such as Parkinson’s disease (PD), microglia-driven neuroinflammation intersects with mitochondrial dysfunction and oxidative stress. PD-associated mutations in nuclear-encoded mitochondrial proteins (e.g., Parkin, PINK1, LRRK2, DJ-1) contribute to impaired mitochondrial homeostasis and are also linked to heightened inflammatory responses. LPS-induced upregulation of G6PD in microglia increases NADPH availability, promotes NF-κB activation and NOX2-dependent oxidative stress, thereby exacerbating dopaminergic neuron degeneration and motor dysfunction. Transcription factors such as IRF5 further modulate these pathways; IRF5 supports pro-inflammatory cytokine expression, whereas its knockdown limits apoptosis-related signaling and enhances antioxidant defenses via Nrf2 and its targets, suggesting a route to attenuate microglia-mediated neurotoxicity [34,178,184,185].

In spinal cord injury (SCI), microglia and blood-derived macrophages are major sources of TNF-α, IL-1β and IL-6, which aggravate secondary damage, glial scar formation and neurological deficits. Although M1-to-M2 conversion can occur during the first week after SCI, this shift is often incomplete. M2-polarized cells can clear necrotic debris and producing anti-inflammatory mediators (TGF-β, IL-10) to create a permissive environment for axonal growth and neuronal survival in experimental rodent models, making enhancement of the M2/M1 ratio an attractive strategy for spinal cord repair. Canonical TLR4/NF-κB signaling drives M1 polarization in this setting: ligand-induced TLR4 activation leads to IκB phosphorylation and degradation, NF-κB nuclear translocation and transcription of pro-inflammatory genes that promote neuronal apoptosis and glial scar formation. Conversely, fibroblast growth factor 4 (FGF4) has been shown to skew microglia/macrophages toward a restorative M2 subtype after SCI, improving functional recovery and axonal regeneration through activation of the PI3K/AKT/GSK3β axis and attenuation of TLR4/NF-κB signaling; pharmacological inhibition of PI3K/AKT/GSK3β or reactivation of TLR4/NF-κB abrogates these beneficial effects [186,187].

Metabolic and ionotropic pathways also interface with neuroinflammation. In CNS diseases, microglia and infiltrating macrophages frequently shift toward pro-inflammatory M1-like profiles, characterized by increased glycolysis and production of NO/ROS and cytokines that exacerbate neuronal injury. N-methyl-D-aspartate receptors (NMDARs), classically known for regulating synaptic plasticity, cognition and neuronal maturation, are expressed in myeloid cells where they can influence macrophage polarization. At the experimental level, the involvement of NMDARs in macrophage polarization has been demonstrated in LPS-stimulated bone marrow-derived macrophages. LPS stimulation upregulates N-methyl-D-aspartate receptor (NMDAR) subunits in parallel with iNOS and NF-κB activation, and NMDAR-dependent Ca2+ influx drives glycolysis, which is required for full M1 polarization. Silencing glycolytic enzymes such as lactate dehydrogenase A (LDHA) reduces extracellular acidification and blunts iNOS induction, underscoring the importance of NMDAR-mediated metabolic reprogramming in M1 programming [188]. In inflammatory conditions, low zinc availability can further enhance NF-κB nuclear translocation and promote a shift toward pro-inflammatory macrophage phenotypes with increased cytokine production, whereas zinc supplementation has been reported to mitigate these responses and improve functional outcomes after CNS injury [29,189].

Emerging evidence also highlights the contribution of stimulator of interferon genes (STING)–type I interferon signaling to microglia/macrophage phenotypic shifts in stroke. Single-cell transcriptomic analyses of ischemic lesions reveal that sustained phagocytosis by microglia and macrophages leads to STING activation, which in turn drives type I interferon responses and promotes a transition toward a more pro-inflammatory state over time. Gene expression trajectories indicate that inflammation-resolving programs predominate in the early to middle stages, whereas pro-inflammatory modules become dominant at later time points. Pharmacological inhibition of STING with H-151 interrupts this phenotypic shift, reduces TNF-α upregulation after DAMPs phagocytosis, preserves phagocytic capacity and attenuates neuroinflammation, while largely sparing IL-10 expression, suggesting that selective modulation of STING may uncouple detrimental inflammation from beneficial clearance functions [176].

Glial–immune crosstalk extends beyond microglia and macrophages. Astrocytes, which outnumber neurons in many CNS regions, rapidly become reactive following ischemic or hemorrhagic insults. Transcriptomic profiling shows that microglia-derived signals can induce a distinct “inflammatory reactive” astrocyte phenotype characterized by upregulation of matrix metalloproteinases such as MMP3, which promotes blood–brain barrier disruption and worsens neurological outcome in hemorrhagic stroke models. Inhibition or astrocyte-specific deletion of MMP3 reduces barrier breakdown and improves functional recovery, underscoring the importance of microglia–astrocyte interactions in neurovascular injury [36].

In demyelinating diseases such as multiple sclerosis (MS), chronic activation of microglia and infiltrating macrophages sustains myelin damage and impairs conduction. Ferroptosis—an iron-dependent, lipid peroxidation-driven form of regulated cell death—has been implicated in MS pathophysiology, with evidence that myelin lipids are more severely affected than proteins. High intracellular iron levels promote M1-like activation of microglia; stimulation with LPS, TNF-α or IL-6 increases expression of iron-handling proteins such as DMT1 and hepcidin, leading to iron accumulation, enhanced TNF-α production and propagation of inflammatory damage. These findings suggest that iron metabolism and ferroptosis pathways are important modulators of neuroinflammation and potential therapeutic targets in demyelinating disorders [64].

The dominant macrophage phenotypes, key molecular mechanisms, and clinical significance identified across the pathological contexts examined in this section are summarized in Table 1.

## 4. Therapy-Driven Reprogramming of Macrophage Plasticity

Therapeutic modulation of macrophage plasticity can occur through two conceptually distinct mechanisms: deliberate, targeted interventions—such as small-molecule polarization switches, engineered macrophage-based therapies, exosome-driven reprogramming, and epigenetic modulators—that directly reprogram macrophage phenotype as a primary objective; and indirect or secondary effects of conventional treatments—including cytotoxic chemotherapy, radiotherapy, and biologics developed for other targets—that reshape macrophage polarization as a collateral consequence of their primary mechanism of action. Both modes of modulation are clinically relevant and are discussed across oncological, autoimmune, inflammatory bowel, infectious, and neurological disease contexts in the subsections below. Metabolic and endocrine organ diseases—including myocardial infarction, obesity-associated adipose inflammation, and diabetic kidney disease—are addressed in detail in §3.6 in terms of macrophage polarization dynamics; dedicated therapeutic strategies for these contexts remain largely preclinical and are therefore not the subject of a separate subsection here. However, challenges related to delivery specificity, off-target effects, and micro-environmental barriers still limit the clinical translation of the most targeted approaches [2,3,5].

Macrophages display a high degree of phenotypic plasticity, making them attractive candidates for therapeutic manipulation across diverse pathological contexts. Rather than representing fixed entities, classically activated (M1-like) and alternatively activated (M2-like) macrophages occupy interconvertible states and can shift between polarization programs in response to evolving microenvironmental cues or to targeted pharmacologic and biologic interventions—a process broadly referred to as macrophage reprogramming. This plasticity underpins therapeutic opportunities not only in oncology, where re-polarizing immunosuppressive TAMs toward a pro-inflammatory state is a central goal, but equally in autoimmune and rheumatic diseases, inflammatory bowel disease, infectious diseases, and neurological conditions, where restoring appropriate macrophage activation is critical for disease control [2,3,4]. In the circulation, flow cytometric quantification of HLA-DR expression on CD14+ monocytes provides a practical readout of systemic macrophage competence: a reduced proportion of HLA-DR+ monocytes reflects both quantitative and qualitative defects in antigen-presenting capacity and has been linked to immunosuppression in both malignant and non-malignant inflammatory settings [190,191].

### 4.1. Oncology

Within tumors, macrophage polarization reflects a dynamic equilibrium rather than discrete, stable M1/M2 categories, and this plasticity can be pharmacologically exploited. Tumor-educated monocytes and macrophages, for example, may produce chemokines such as CXCL12 that facilitate the transition of M1-polarized cells toward immunosuppressive M2-like phenotypes, thereby reinforcing tumor-promoting circuits. Conversely, targeting key signaling nodes—including PI3K-γ, CSF-1/CSF-1R, STAT3 and STAT6—can reorient M2-skewed TAMs toward a more pro-inflammatory, M1-like profile, supporting the concept of therapeutic “reprogramming” rather than simple depletion. Single-cell transcriptomic analyses in breast cancer further underscore TAM heterogeneity, revealing multiple PD-L1+ TAM subsets, some preferentially enriched in estrogen receptor–negative disease, with potential implications for tailoring immunomodulatory strategies [192,193,194,195].

Macrophages also influence responsiveness to PD-1/PD-L1 checkpoint blockade. In adaptive resistance, tumor cells upregulate PD-L1 to engage PD-1 on effector T cells and dampen cytotoxic activity, while TAMs contribute by fostering dysfunctional or exhausted T-cell states and by amplifying PD-L1 expression on malignant cells and other immunosuppressive leukocytes, thereby reinforcing local checkpoint signaling and promoting resistance to anti-PD-1/PD-L1 therapies. In parallel, several macrophage-specific checkpoints have been defined that functionally mirror T-cell checkpoints. The CD47–SIRPα axis delivers a potent “don’t-eat-me” signal that inhibits macrophage phagocytosis of tumor cells; blockade of CD47 or SIRPα enhances tumor cell clearance, augments antibody-dependent cellular cytotoxicity and can synergize with T-cell-directed immunotherapies [135]. A related pathway involves CD24 on tumor cells and Siglec-10 on macrophages, and disrupting this interaction similarly restores macrophage-mediated phagocytosis and sensitizes tumors to immunotherapy. In addition, TREM2+ TAMs have emerged as a highly immunosuppressive subset enriched in tumors that are refractory to checkpoint inhibition; in preclinical models, TREM2 blockade enhances CD8+ T-cell infiltration and converts “cold” tumors into more inflamed, therapy-responsive lesions. LILRB2 (ILT4) represents another inhibitory receptor expressed at high levels on CD163+ TAMs, and blocking LILRB2 signaling has been shown to relieve myeloid suppression and promote antitumor immunity [44,45,196].

Epigenetic mechanisms provide an additional layer of control over TAM function. Across solid malignancies such as breast and lung cancer, gliomas and hepatocellular carcinoma, histone methyltransferases (for example, EZH2), aberrant DNA methylation and sirtuin deacetylases (notably SIRT1 and SIRT6) have been implicated in skewing macrophage transcriptional programs toward either pro-inflammatory M1-like or immunosuppressive M2-like states, thereby shaping the balance between effective immune surveillance and tumor tolerance [14,197].

Beyond soluble targeting, cell-based strategies have expanded the macrophage-directed therapeutic repertoire. Chimeric antigen receptor-engineered macrophages (CAR-M) are modified to express tumor-specific CARs, enabling selective recognition and phagocytosis of cancer cells while concurrently releasing inflammatory mediators and presenting tumor antigens to T cells. Proof-of-concept approaches include macrophages engineered to secrete IL-12 within the tumor microenvironment, thereby boosting local cytotoxic T-cell activity and reinforcing a pro-inflammatory milieu. In parallel, macrophage depletion strategies using Chimeric Antigen Receptor T (CAR-T) cells directed against TAM-restricted markers such as CD163 have been explored; these CAR-T cells eliminate protumor macrophages, secrete IFN-γ and indirectly reactivate antitumor immunity, offering a targeted means of debulking immunosuppressive myeloid populations while sparing other lineages [198,199,200].

Macrophage-derived extracellular vesicles (EVs) represent a complementary cell-free modality. In contrast to tumor-derived EVs, which often promote myeloid recruitment and immune suppression, ex vivo-engineered macrophage EVs can be loaded with proteins, siRNAs, miRNAs or mRNAs to modulate immune or tumor cells within the tumor microenvironment [201]. Macrophages can also serve as “Trojan horses” for drug delivery: macrophage–drug conjugates exploit the natural tumor-homing properties of these cells to transport therapeutic payloads into poorly perfused or otherwise inaccessible tumor regions [202].

Despite significant advances, important challenges remain. TAM plasticity allows even successfully reprogrammed macrophages to revert to immunosuppressive states under persistent tumor-derived signals, and commonly used M2 markers such as CD206 and CD163 are also expressed on non-tumoral macrophage subsets (for example, liver sinusoidal macrophages), raising concerns about off-target effects. Moreover, TAM-targeted monotherapies frequently yield modest benefit, as tumors can compensate by recruiting alternative suppressive myeloid populations or by activating redundant chemokine and growth factor pathways to restore an immunosuppressive niche. From a translational standpoint, the engineering, persistence and delivery of macrophage-based products—including CAR-M and immortalized macrophage lines—remain technically demanding, and optimal combinations with T-cell-directed immunotherapies and other modalities are still being defined [198,199,203].

Macrophages occupy a central position at the intersection of metastatic progression and therapeutic response. In bone-metastatic breast cancer, for example, CD185-expressing myeloid cells accumulate in the skeletal niche, where CD137 signaling supports monocyte/macrophage recruitment and osteoclast differentiation, thereby promoting osteolytic lesion formation; liposomal anti-CD137 antibodies markedly reduce bone metastases in vivo in preclinical models. In the lung, tissue-resident and infiltrating macrophages are similarly indispensable for metastatic seeding and outgrowth [51]. In MMTV-PyMT mammary carcinoma, combining cytotoxic chemotherapy or PD-1 blockade with histone deacetylase (HDAC) inhibitors reprograms lung-infiltrating macrophages toward a pro-inflammatory, immunostimulatory phenotype and improves antitumor efficacy compared with single-agent chemotherapy or checkpoint inhibition [204]. Consistent with this, in osteosarcoma lung metastasis models, PD-1 blockade reduces metastatic burden in part by shifting TAMs from an M2-biased toward an M1-like state [205].

Macrophage-intrinsic metabolism has emerged as a powerful lever for reprogramming. Inhibition of glutamine synthetase, the enzyme that converts glutamate to glutamine, skews M2-polarized TAMs toward an M1-like phenotype characterized by enhanced glycolysis and HIF-1α activation, translating into reduced metastasis, impaired angiogenesis and attenuation of immunosuppression in vivo in murine tumor models [206]. At the same time, standard chemotherapy can paradoxically foster metastasis by reshaping myelopoiesis and the myeloid compartment. Several cytotoxic regimens drive reactive myelopoiesis biased toward monocyte production, increase perivascular macrophage density and promote tumor revascularization and relapse. In murine lung cancer models, this involves chemotherapy-induced mitochondrial ROS in bone marrow progenitors, elevated CCL2 production and upregulation of coagulation factor X in lung interstitial macrophages, together coupling coagulation, inflammation and monocyte/macrophage recruitment to a pro-metastatic lung niche [207].

Parallel transcription programs in tumor cells can feed back on macrophage behavior. In breast cancer, low SOCS2 expression in tumor tissue is associated with increased M2 polarization and more aggressive disease. Overexpression of CEBPA in tumor cells upregulates SOCS2, limits M2 skewing, and suppresses tumor growth, angiogenesis and metastasis; silencing SOCS2 abrogates these CEBPA-mediated effects, indicating that SOCS2 is a critical mediator of CEBPA-driven macrophage modulation and antitumor activity. Notably, SOCS2 functions in a context-dependent manner across cancer types: it can enhance radiosensitivity and ferroptosis in hepatocellular carcinoma by promoting SLC7A11 ubiquitination, is downregulated by METTL3 to support colon cancer growth, and may act as a pro-tumor factor in acute myeloid leukemia and prostate cancer. This duality mirrors the functional heterogeneity of macrophages and underscores how the same molecular node can be protective or deleterious depending on tissue and oncogenic context [208,209,210,211,212,213].

Cytotoxic chemotherapy itself is a potent driver of extracellular vesicle (EV) release. Tumor cells exposed to agents such as paclitaxel or doxorubicin shed abundant EVs that can reprogram the tumor microenvironment, promote immune escape and facilitate metastatic conditioning even as primary tumors shrink, reinforcing the view of EVs as key intermediates in chemoresistance and pre-metastatic niche formation [214,215].

In pancreatic ductal adenocarcinoma (PDAC), stromal–myeloid crosstalk exemplifies how macrophages shape T-helper polarization. Cancer-associated fibroblast (CAF)-derived thymic stromal lymphopoietin (TSLP), induced by inflammasome-dependent IL-1β signals from macrophages, promotes Th2-skewing dendritic cells and biases macrophages toward M2-like phenotypes, consolidating a Th2-dominant, immunosuppressive milieu. Pharmacologic IL-1 receptor blockade with agents such as anakinra reduces TSLP production by CAFs in vitro and in murine PDAC models in vivo, illustrating how targeting upstream inflammasome–IL-1 signaling can indirectly modulate both macrophage and T-helper cell polarization [216,217].

In the CNS, radiotherapy (RT) acts as an indirect macrophage modulator: by inducing immunogenic cell death and releasing DAMPs (phosphatidylserine, calreticulin, HMGB1), RT primes the myeloid compartment for enhanced phagocytic activity. Combining RT with disruption of the CD47–SIRPα phagocytic checkpoint yields synergistic myeloid activation and may overcome phagocytic resistance in diffuse midline glioma and glioblastoma—illustrating how conventional radiotherapy can potentiate macrophage-directed immunotherapy [218].

A growing body of work now focuses on deliberate macrophage reprogramming as a therapeutic strategy, through either small molecules or engineered products. At the pharmacologic level, the antiangiogenic agent 2-methoxyestradiol (2ME2) illustrates selective targeting of M2 polarization. In preclinical breast cancer models, low-dose 2ME2 disrupts microtubules, reduces CD206 and CD163 expression and inhibits a broad set of M2-associated mediators—including CCL18, TGF-β, IL-10, arginase, CXCL12, MMP9 and VEGF-A—without significantly affecting M1 polarization. These effects correlate with reduced STAT3 phosphorylation and nuclear localization and can be reversed by STAT3 activation, supporting a mechanism in which 2ME2 suppresses protumoral macrophage functions via STAT3 inhibition [219].

At the nucleic acid and vesicle interface, exosome-based antisense approaches have been developed to directly reprogram M2 macrophages. Engineered exosomes carrying antisense oligonucleotides against STAT6 or C/EBPβ (exoASO-STAT6 and exoASO-C/EBPβ) selectively deliver these cargos to M2-like macrophages, silence the corresponding transcription factors, and convert TAMs into M1-like, pro-inflammatory effectors. In aggressive orthotopic hepatocellular carcinoma models, systemic administration of such exosome–ASO constructs achieves robust tumor control, demonstrating the feasibility of macrophage-focused exosome therapeutics [220].

Immortalized macrophage lines provide another platform to exploit macrophage biology. Retroviral (J2) immortalization of bone marrow-derived macrophages (iBMDMs) and other tissue macrophages yields proliferative lines that retain the capacity to polarize toward M1 or M2 states and are amenable to genetic engineering and mechanistic interrogation [221]. Under M1 conditions, iBMDMs secrete high levels of TNF-α and IL-12, low levels of IL-10 and TGF-β, and display strong phagocytic activity, often exceeding that of primary BMDMs and immortalized lines such as RAW264.7; supernatants from M1-iBMDMs upregulate pro-apoptotic genes (e.g., APAF1, caspase-9) and downregulate BCL2 in tumor cells, consistent with TNF-α-mediated cytotoxicity. Clinical translation is currently limited by the lack of human immortalized macrophages suitable for therapy, but insights from iBMDM systems may inform strategies to immortalize other innate effectors (NK cells, dendritic cells, T cells) for gene-edited, off-the-shelf immunotherapies [222,223,224].

Finally, macrophages intersect with broader T-cell-centric approaches. Effective immunotherapy requires both robust priming of tumor-specific cytotoxic T lymphocytes (CTLs) and active remodeling of the tumor microenvironment to relieve myeloid and regulatory constraints. “Cold” tumors with poor cytotoxic T lymphocyte (CTL) infiltration respond poorly to vaccines, checkpoint blockade and adoptive T-cell therapy, in part because of the accumulation of immunosuppressive Treg cells, MDSCs and TAMs that exclude CTLs from the tumor bed and inhibit their TNF-α and IFN-γ production. Dendritic cells are central to this process as the most potent antigen-presenting cells: robust T-cell priming requires adequate pattern-recognition receptor stimulation, whereas antigen presentation by immature or insufficiently activated dendritic cells promotes T-cell anergy and tolerance. Rational vaccine design has incorporated these principles. Lipid-glycopolymer-conjugated CpG formulated on gold nanoparticles (LCpG) has been engineered to preferentially target lymphoid tissues, where it drives strong Tc1 responses; in tumor-bearing mice, LCpG-based vaccination enhances antigen-specific CD8+ T-cell infiltration into the tumor, increases CTL-derived IFN-γ, reshapes the myeloid compartment and limits lung metastases. When combined with checkpoint blockade, such vaccines show synergistic activity, illustrating how coordinated modulation of DC activation, CTL priming and the suppressive myeloid milieu—particularly TAMs—can convert immunologically refractory tumors into responsive lesions [225,226,227].

In pancreatic ductal adenocarcinoma (PDAC), macrophages are key regulators of how tumors respond to chemotherapy and immunotherapy. Chemotherapeutic regimens in orthotopic PDAC models can paradoxically increase the likelihood that residual tumor cells survive; this chemoprotective effect is substantially reduced when intratumoral macrophages are depleted or when recruitment of CCR2+ inflammatory monocytes is inhibited. Mechanistically, tumor-associated macrophages (TAMs) supply insulin-like growth factors 1 and 2 that attenuate gemcitabine cytotoxicity, and macrophages and fibroblasts are major sources of IGFs within the PDAC stroma [228]. Combining gemcitabine with IGF-neutralizing strategies improves tumor control in preclinical models, producing smaller tumors and higher levels of tumor cell death than chemotherapy alone [229]. In mammary tumor models, chemotherapy also increases macrophage infiltration and upregulates cathepsin proteases in these cells; cathepsin-expressing macrophages protect neighboring cancer cells from taxane- and anthracycline-induced apoptosis, whereas pharmacologic cathepsin inhibition restores sensitivity and enhances the efficacy of multiple cytotoxic agents [230].

Therapeutic approaches that diminish TAM abundance or function can therefore mitigate immune escape and treatment resistance. Blocking CSF-1R signaling reduces the TAM pool by interfering with both monocyte-to-macrophage differentiation and with the survival of established TAMs, and in PDAC models, CSF-1R inhibition preferentially depletes immunosuppressive CD206^hi subsets, enhances antigen-presenting features and improves CD8+ T-cell-mediated immunity, particularly when combined with checkpoint inhibitors [231]. In parallel, targeting chemokine pathways limits ongoing myeloid recruitment: PDACs produce high levels of CCL2 and are infiltrated by CCR2+ immunosuppressive macrophages, and clinical as well as preclinical data indicate that blocking the CCL2/CCR2 axis depletes inflammatory monocytes and macrophages from primary tumors and premetastatic sites, reduces tumor growth and metastasis, and enhances antitumor T-cell responses [228,232].

The CCL2/CCR2 axis already represents an emerging therapeutic target, but its efficacy may be constrained by chemokine redundancy and compensatory pathways, thereby underscoring the need for combination strategies and a deeper understanding of organ-specific mechanisms [233].

More selective strategies focus on receptors enriched in immunosuppressive TAM subsets. The scavenger receptor MARCO is preferentially expressed on M2-skewed macrophages in the tumor microenvironment and is associated with an immunosuppressive transcriptional program, higher IL-37 levels and expansion of regulatory T cells. In pancreatic cancer, tumor tissues exhibit higher MARCO and CD163 expression than adjacent non-tumor tissue, with substantial inter-patient heterogeneity; high expression of either marker independently correlates with worse overall and disease-free survival, suggesting that CD163+/MARCO+ TAMs have prognostic relevance and may represent actionable targets [57].

Macrophages can also be converted into active therapeutic tools through chimeric antigen receptor engineering. CAR-modified macrophages (CAR-M) directed against tumor- or myeloid-associated antigens such as CD47 or CSF-1R can recognize and engulf cancer cells while simultaneously secreting pro-inflammatory mediators, recruiting additional immune effectors and presenting tumor antigens to T cells, thereby linking direct phagocytosis with broader remodeling of the tumor immune microenvironment [45].

Finally, macrophage metabolism offers additional, highly specific points of intervention. Arginase-1, abundantly expressed in M2-like TAMs, depletes extracellular arginine and contributes to T-cell dysfunction in PDAC. In a dual-recombinase genetically engineered PDAC model, macrophage-specific deletion of Arg1 delays invasive disease and is accompanied by increased CD8+ T-cell infiltration. Pharmacologic arginase inhibition with CB-1158 in established tumors further augments CD8+ T-cell recruitment beyond that achieved with genetic Arg1 loss alone and sensitizes tumors to anti-PD-1 checkpoint blockade, positioning Arg1 as a promising immunometabolic target to enhance immunotherapy efficacy in pancreatic cancer [95].

In acute myeloid leukemia (AML), macrophages are increasingly recognized as active contributors to chemoresistance. NIPA1 (Prader–Willi/Angelman syndrome 1) has recently been identified as a macrophage-linked determinant of treatment response: it is overexpressed in AML, and its high expression associates with poorer overall survival. Experimental depletion of NIPA1 in IL-4/IL-13-polarized M2-like macrophages attenuate IGFBP2/EGFR signaling, weakens leukemia cell survival and reduces anthracycline resistance. In vitro, NIPA1 knockdown in TAMs increases CD86 and decreases CD163 expression, indicating a shift toward an M1-like phenotype, and in vivo in murine AML models this is mirrored by increased CD86+ and reduced CD163+ macrophage infiltration in leukemic tissues. Functionally, TAM-targeted NIPA1 depletion enhances chemosensitivity and slows disease progression in both parental and adriamycin-resistant AML models, pointing to NIPA1 as a potential immunotherapeutic target in myeloid leukemia [234].

In B-cell malignancies, antibody-based platforms that engage both T cells and myeloid cells are gaining prominence. Bispecific antibodies directed against CD19 or CD20 on B cells and CD3 on T cells (such as blinatumomab, epcoritamab and TNB-486) recruit cytotoxic T cells to malignant B cells and can secondarily activate macrophages within the lymphoma microenvironment. Although these agents have shown meaningful activity, their efficacy as monotherapies is often limited, underscoring the need for rational combinations and biomarker-guided patient selection [45].

Nanotechnology offers highly selective tools for modulating TAMs in vivo. Manganese dioxide-based nanoparticles, for instance, can relieve tumor hypoxia—a driver of M2 polarization—while promoting M1 reprogramming, thereby enhancing TAM-mediated tumor killing [235]. Metabolic vulnerabilities can also be exploited: inhibition of CPT1A, a key enzyme in fatty acid oxidation, preferentially impairs M2-like TAMs, which depend heavily on this pathway, reducing tumor growth in preclinical models while sparing other myeloid subsets [236]. Mannose-decorated nanoparticles that bind CD206 on M2 TAMs enable targeted delivery of cytotoxic drugs such as doxorubicin directly into immunosuppressive macrophages, achieving local TAM depletion with reduced systemic toxicity [237].

Small activating RNAs like MTL-CEBPA upregulate CEBPA, a transcription factor central to myeloid differentiation and macrophage polarization; early clinical data indicate that CEBPA activation can reorient myeloid cells toward more immunostimulatory phenotypes and sensitize tumors to conventional treatments. Circular RNAs, by virtue of their covalent closed structure and enhanced stability, are being explored as durable modulators of macrophage gene expression, although this area remains nascent [238].

Leukemia-intrinsic pathways can also dictate myeloid composition. In T-ALL, stromal interaction molecules STIM1 and STIM2 in malignant T cells control store-operated calcium entry and drive cancer-induced inflammation. Genetic ablation of Stim1/Stim2 in leukemic cells reduces necroinflammatory responses, diminishes macrophage infiltration and restores a more pro-inflammatory TAM phenotype via interferon-dependent mechanisms, highlighting leukemic calcium signaling as an upstream regulator of myeloid recruitment and functional polarization [239].

Several macrophage-targeted interventions have been evaluated directly in hematologic malignancies. In CLL, pharmacologic CSF-1R inhibition with agents such as pacritinib depletes lymphoma-associated macrophages and reduces leukemic cell viability, supporting CSF-1R as a key component of the CLL myeloid niche [240]. In follicular lymphoma, tumor-derived CSF-1 promotes M2-like macrophage skewing, and the CSF-1R inhibitor pexidartinib (PLX3397) preferentially affects M2 macrophages, repolarizing TAMs toward an M1-like phenotype and cooperating with anti-CD20 rituximab to achieve superior antitumor effects [241].

Myeloid checkpoints such as CD47–SIRPα are also being actively explored in blood cancers. Blocking CD47 on malignant hematopoietic cells in AML, CLL and multiple myeloma enhances macrophage phagocytosis and, via improved dendritic-cell cross-priming, promotes T-cell-mediated tumor rejection [242,243,244]. A bispecific antibody co-targeting CD47 and CD20 recapitulates and amplifies the synergy seen with rituximab plus anti-CD47 in non-Hodgkin lymphoma models, significantly improving survival [243,245].

Macrophage migration inhibitory factor (MIF) represents another myeloid-centered target in AML. MIF is highly expressed by AML blasts and elevated in patient sera, and pharmacologic inhibition of MIF can shift macrophages toward an M1-like phenotype, especially when combined with pro-inflammatory cytokines and CSF-1R blockade. In preclinical models combining GM-CSF with MIF inhibitors reprograms M2 macrophages into M1, increases leukemic cell apoptosis and reverses resistance to FLT3 and BCL-2–directed therapies; in xenografts, this strategy reduces leukemia burden, although it has yet to be tested in clinical trials [246].

Finally, bisphosphonates exemplify how drugs developed for bone disease can impact tumor–macrophage interactions. Both non-nitrogenous and nitrogen-containing bisphosphonates inhibit tumor cell proliferation, induce apoptosis, interfere with angiogenesis and alter immune surveillance, while also suppressing macrophage proliferation, migration and invasion and ultimately inducing macrophage apoptosis. Zoledronate, a third-generation nitrogen-containing bisphosphonate, shows selective cytotoxicity toward MMP9-expressing TAMs and has been reported to restrain cervical cancer progression in preclinical studies, suggesting that bone-targeted agents can be repurposed to selectively target protumoral macrophages in solid tumors [3,38].

In melanoma, macrophage-directed strategies have focused on both TAM depletion and phenotypic reprogramming. CSF-1R inhibitors such as PLX3397 reduce TAM abundance in the melanoma microenvironment, while anti-MARCO antibodies convert immunosuppressive M2-like macrophages toward an M1-like, pro-inflammatory state, enhancing the efficacy of anti-cytotoxic T-lymphocyte-associated protein 4 (CTLA-4) checkpoint immunotherapy in preclinical models. Complementary approaches include TLR agonists such as imiquimod, which promote M1 activation, and innate immune checkpoint inhibitors targeting the CD47/SIRPα axis, which restore macrophage phagocytic capacity and reduce tumor growth in preclinical settings [247,248].

### 4.2. Autoimmune and Rheumatic Diseases

For rheumatic diseases, metformin, a widely used antidiabetic drug, has been shown to inhibit M1 polarization of synovial macrophages and reduce pro-inflammatory cytokine production, thereby attenuating cartilage damage in experimental osteoarthritis models. Mitogen-activated protein kinase phosphatase-1 (MKP-1), a key negative regulator of mitogen-activated protein kinase (MAPK) signaling, similarly influences macrophage fate: MKP-1 not only limits pro-inflammatory activation but actively promotes alternative, anti-inflammatory polarization, and glucocorticoids achieve part of their effect by engaging MKP-1 and shifting macrophages from M1- toward M2-like states. This ability of glucocorticoids to drive M2c-type, pro-fibrotic macrophage differentiation may help explain their unfavorable impact in fibrosing conditions such as systemic sclerosis [106,249,250].

A range of anti-inflammatory and biologic therapies modulate macrophage signaling. TNF inhibitors (e.g., infliximab, etanercept, adalimumab, golimumab, certolizumab) dampen key intracellular pathways in monocyte-derived macrophages, including NF-κB and STAT3-centered cascades, and can normalize aberrant SOCS3 and GAS6 signaling in M1-like macrophages from patients with rheumatoid arthritis (RA). More broadly, suppressor of cytokine signaling (SOCS) proteins regulate multiple cytokine and growth factor pathways downstream of JAK–STAT, and dysregulated SOCS expression has been linked to chronic inflammation and malignancy. In systemic sclerosis (SSc), standard therapies such as vasodilators (e.g., bosentan) and immunosuppressants (glucocorticoids, tocilizumab, JAK inhibitors) exert substantial effects on tissue macrophages, while antifibrotic agents such as nintedanib may also limit organ damage by preventing the emergence of pro-fibrotic macrophage phenotypes [100,101,102,106,108,109,110].

Macrophage phenotype is an important determinant of treatment response in systemic autoimmunity. In lupus nephritis, a high density of CD68+ macrophages in the tubulointerstitial predicts poor response to immunosuppressive therapy, and transcriptomic studies show upregulation of pro-inflammatory genes such as LGALS9 in renal macrophages from patients compared with healthy controls. Although pro-fibrotic macrophage phenotypes are often associated with the resolution of renal inflammation, they may also promote maladaptive scarring. Mycophenolate mofetil (MMF) and its active metabolite mycophenolic acid, widely used in renal and non-renal systemic lupus erythematosus (SLE), appear to influence monocyte/macrophage plasticity [251,252]. In lupus-prone mice, dextran-based mycophenolate nanoparticles accumulate in kidney and spleen macrophages and promote a shift toward an anti-inflammatory M2-like phenotype, characterized by increased CD206 and reduced CD80/CD40 expression and diminished TNF-α production, paralleling the observed amelioration of kidney injury [253].

In large-vessel vasculitis such as giant cell arteritis (GCA), glucocorticoids selectively reduce circulating non-classical monocytes, whereas classical and intermediate subsets persist. Macrophages in temporal artery lesions from GCA patients commonly express CD16 and CX3CR1, consistent with a derivation from non-classical monocytes, and this subset appears to be less effectively targeted by current therapies. Mavrilimumab, a monoclonal antibody against the GM-CSF receptor α chain, has demonstrated efficacy in reducing vascular inflammation in GCA, further highlighting the therapeutic value of modulating monocyte–macrophage axes in vasculitis [110].

Several regulatory pathways have been identified that directly modulate macrophage polarization and offer novel therapeutic opportunities in rheumatic disease. IL-35 promotes apoptosis of inflammatory fibroblast-like synoviocytes, suppresses TNF-α-driven M1 macrophage activation and enhances alternative M2 polarization, thereby ameliorating collagen-induced arthritis in vivo [254]. IL-38 reduces the production of M1-associated cytokines and dampens Th17 responses, supporting a shift toward a less inflammatory macrophage phenotype in experimental arthritis [255]. Activation of the nuclear receptor NR1D1 (REV-ERBα), a transcriptional repressor implicated in circadian and metabolic control, decreases inflammatory cytokine production by RA fibroblasts and blocks M1 polarization by inhibiting MAPK and NF-κB signaling, suggesting that NR1D1 agonists could serve as anti-inflammatory agents in RA [256]. Triggering receptor expressed on myeloid cells-2 (TREM2), expressed on macrophages and dendritic cells, has also been implicated in promoting M2-like polarization in several disease models; in osteoarthritis, TREM2 activation facilitates the transition from M1 to M2 via the NF-κB/CXCL3 axis and alleviates joint pathology in mice [257].

### 4.3. Inflammatory Bowel Disease and Skin Inflammation

Mesenchymal stem cells (MSCs) and their soluble mediators can modulate intestinal inflammation in part by acting on macrophages. In experimental IBD, MSC-derived exosomes and tumor necrosis factor-α-stimulated gene/protein 6 (TSG-6) promote a switch of infiltrating macrophages toward an M2-like phenotype, reduce pro-inflammatory cytokine production in the colon and ameliorate colitis severity, highlighting M2 polarization as a key component of the protective effect of MSC-based therapies. TNF-α, which is markedly increased in the inflamed mucosa of IBD patients and produced by multiple cell types (including macrophages, adipocytes, fibroblasts and T cells), plays a central pathogenic role, as underscored by the efficacy of anti-TNF agents; dysregulated TNF-α levels, in concert with elevated IL-17, IL-21, IL-22 and IL-9, contribute to the breakdown of mucosal homeostasis and drive chronic intestinal inflammation. Paradoxically, anti-TNF monoclonal antibodies can also trigger immune-mediated adverse events—such as psoriasiform skin lesions, arthritis, autoantibody production and, rarely, lupus-like syndromes and vasculitis—which typically resolve upon treatment discontinuation and are thought to reflect imbalances between pro-inflammatory and regulatory cytokine networks [121,258].

A substantial accumulation of macrophages is also a hallmark of atopic dermatitis (AD), where M2-like cells appear to be particularly important in shaping disease activity and responses to therapy. Natural flavonoids have emerged as potential modulators of macrophage behavior in this context. Naringenin has been shown in NC/Nga mouse models of AD to suppress pro-inflammatory M1-like macrophages and accelerate their reprogramming toward an M2-oriented phenotype, leading to reduced skin inflammation and improved barrier function. Diosmetin, via a distinct mechanism, decreases macrophage infiltration into dinitrochlorobenzene-induced AD lesions and lowers local cytokine levels, thereby diminishing epidermal and dermal thickening and overall disease severity. Together, these findings illustrate how targeting macrophage polarization and recruitment—whether through MSC-derived factors in IBD or plant-derived flavonoids in AD—can help restore tissue homeostasis across diverse inflammatory skin and gut diseases [259,260].

### 4.4. Infectious Diseases

The recent literature highlights a rapidly expanding repertoire of strategies aimed at modulating macrophage death pathways, metabolism, polarization, and tissue residency to improve infection outcomes [159,261].

One emerging approach involves the regulation of macrophage pyroptosis, a highly inflammatory form of programmed cell death. Ophiopogonin C has been shown to attenuate pyroptosis in murine models of sepsis-induced acute lung injury. Mechanistically, the molecule interferes with the DDX3X–NLRP3 axis, reducing inflammasome activation and limiting tissue damage. These findings underscore the therapeutic potential of targeting pyroptotic pathways to mitigate systemic inflammation and organ injury during severe infections [262].

Metabolic rewiring has also emerged as a promising target for macrophage-centered therapies, particularly in chronic infections. Many pathogens reshape macrophage glycolytic, TCA-cycle, and lipid-oxidation pathways to maintain an immunoregulatory, M2-skewed state. Pharmacological strategies that re-establish M1-linked metabolic programs—through modulation of succinate, itaconate, or HIF-1α signaling—may help reverse pathogen-induced immune suppression and enhance antimicrobial efficacy [263].

MST1 (mammalian STE20-like kinase 1) represents a promising therapeutic target in parasitic infections. In schistosomiasis models, pharmacological activation of MST1 enhances PPARγ-dependent phagocytosis and promotes antifibrotic macrophage polarization, reducing hepatic granuloma formation and liver fibrosis, and positioning MST1 agonism as a candidate strategy for M2-driven fibrotic disease [165].

Innovative biomaterial-based strategies have also been developed to guide macrophage behavior in infected tissues. Injectable immunoregulatory hydrogels were engineered to induce an early M1 response for bacterial clearance, followed by a transition to a regenerative M2 phenotype to support wound healing. This sequential modulation of macrophage polarization demonstrates how smart biomaterials can be leveraged to coordinate immune defense and tissue repair in cutaneous infections [264].

Pathogen-derived extracellular vesicles represent an emerging target for macrophage-directed intervention. In Helicobacter pylori infection, CagA-carrying exosomes activate the JAK/STAT1 axis and trigger macrophage ferroptosis; restoring SLC7A11-mediated ferroptosis resistance reduces gastric inflammation, suggesting that limiting pathogen-induced ferroptotic immunopathology is a therapeutically actionable strategy in bacterial gastric disease [161].

Beyond direct signaling manipulation, trafficking of resident macrophage subsets represents an underexplored therapeutic lever. Pleural macrophages that migrate into the lung parenchyma during influenza contribute to viral clearance and tissue repair; depletion experiments show that their loss worsens disease severity, suggesting that strategies to sustain or amplify their pulmonary recruitment could improve outcomes in respiratory infections [163].

Macrophage migration inhibitory factor (MIF) and its homolog D-DT also emerge as context-dependent immunomodulatory targets. Depending on the infectious setting, MIF can either support protective immunity or amplify pathological inflammation. A range of inhibitors—including neutralizing antibodies, small-molecule tautomerase blockers, and peptide-based antagonists—is currently under investigation for conditions such as sepsis, reflecting the complex and dualistic nature of MIF-mediated signaling [30,159,173].

Several therapeutic approaches act indirectly by shaping macrophage activation states. mRNA-based vaccines, such as mRNA-1273, are efficiently taken up by skin-resident dendritic cells and macrophages, triggering maturation pathways and highlighting these cells as natural targets for nucleic-acid-based immunization strategies. In contrast, macrophage dysfunction can worsen disease severity, as illustrated during SARS-CoV-2 infection: the uptake of virus-infected apoptotic cells disrupts efferocytosis and drives macrophages toward a pro-inflammatory program marked by elevated IL-6 and IL-1β production. Restoring effective efferocytic activity may therefore represent a therapeutic avenue to limit immunopathology in viral infections [265,266].

Taken together, current findings indicate that diverse strategies aimed at reprogramming macrophage activity—ranging from modulation of cell-death pathways and metabolic states to MST1-dependent polarization, ferroptotic control, efferocytic enhancement, and the use of biomaterial-based approaches—are rapidly expanding the therapeutic landscape. Despite their heterogeneity, these interventions converge on a central principle: fine-tuning macrophage functional programs can markedly alter the course of infectious diseases and may underpin the development of next-generation immunomodulatory treatments [159,165,261,262,265].

Within the context of macrophage-targeted antifungal therapies, emerging evidence highlights TREM2 as a promising intervention point. Modulating this receptor can partially restore antifungal competence in CARD9-deficient macrophages, enhancing their effector functions and improving fungal control in experimental models. These findings position TREM2 as a druggable checkpoint whose regulation—together with upstream pathways such as the NF-κB/CREB axis—may offer a viable strategy for correcting macrophage dysfunction in chronic dematiaceous fungal infections associated with CARD9-related immunodeficiency [174].

### 4.5. Neurological Diseases

In neurodegenerative diseases, like Parkinson’s disease, metabolic reprogramming of microglia toward a glycolytic, M1-like state has been implicated in dopaminergic neuron loss, and experimental studies in murine models show that inhibiting specific metabolic pathways in M1 microglia or promoting a shift toward an M2-like phenotype can attenuate neuroinflammation and protect nigrostriatal neurons [34].

DAMPs such as high mobility group box 1 (HMGB1) are important upstream regulators of microglia/macrophage activation in acute CNS injury. In spinal cord injury (SCI) models, intraspinal HMGB1 injection induces upregulation of CD86, TNF-α, IL-1β and iNOS in local microglia/macrophages, consistent with a transition to a neurotoxic M1 phenotype [267,268]. Conversely, biomaterial-based approaches that neutralize DAMPs can favor repair: a DAMP-scavenging, IL-10-releasing hydrogel reduces HMGB1/RAGE signaling after SCI, increases M2 skewing of microglia/macrophages and is associated with axonal regrowth and improved motor recovery [269]. Similar observations with other alarmins (e.g., IL-33, extracellular nucleotides, heme) indicate that dampening danger-signal pathways promotes the emergence of anti-inflammatory M2 cytokines and supports resolution of post-traumatic inflammation [270,271].

Pro- and anti-inflammatory signaling cascades converge on shared intracellular hubs that govern myeloid phenotype in the injured CNS. In spinal cord injury (SCI), inhibition of the canonical TLR4/NF-κB pathway reduces inflammatory cell infiltration and pro-inflammatory cytokine production, thereby facilitating axonal regrowth and functional recovery. Recombinant FGF4 (rFGF4) provides a concrete example of how these pathways can be harnessed therapeutically: in experimental SCI, rFGF4 promotes neural regeneration and locomotor recovery while skewing microglia/macrophages toward an M2-like phenotype in vivo, in part through PI3K/AKT/GSK3β activation and attenuation of TLR4/NF-κB signaling [186].

Together, these findings reinforce the notion that microglia and macrophages integrate inflammatory, metabolic and epigenetic cues to adopt distinct functional states across CNS pathologies—from acute trauma and stroke to chronic neurodegeneration and brain tumors. Therapeutic strategies that selectively reprogram these cells, rather than broadly suppressing inflammation, offer a promising route to limit neurotoxicity, preserve endogenous repair mechanisms and improve outcomes across diverse neurological disorders [2,15,34,119].

The main therapeutic strategies targeting macrophage plasticity discussed in this section, together with their mechanistic targets and key preclinical and clinical findings, are summarized in Table 2.

## 5. Discussion

The body of evidence synthesized in this review delineates a set of converging mechanisms that collectively reshape the biological landscape of these highly complex cells, the macrophages. In this Discussion, we move beyond descriptive summaries to integrate current findings, compare emerging conceptual models, and articulate the broader implications that arise from recent advances in the field.

A central objective of this review is to elucidate how macrophage plasticity differs across anatomical sites, shaped by local microenvironmental cues, interacting with cellular networks, and disease-specific contexts. The conditions selected for in-depth analysis were identified according to three predefined criteria—recent scientific output, clinical prevalence, and disease aggressiveness—to ensure the inclusion of representative and clinically relevant scenarios. The literature search was conducted in PubMed/MEDLINE using the following core search terms: “macrophage polarization”, “macrophage plasticity”, “M1/M2 macrophage”, “tumor-associated macrophage”, “monocyte phenotype”, and “flow cytometry”, combined with disease-specific terms for each section (e.g., “systemic sclerosis”, “glioblastoma”, “polymyalgia rheumatica”, “Parkinson’s disease”). The search was restricted to publications in English from January 2019 to April 2026, except for seminal earlier works cited for mechanistic context. Reference lists of retrieved articles were also screened for additional relevant publications. No formal systematic review protocol was registered; this article is intended as a narrative review. During the preparation of this manuscript, the author used Claude Sonnet 4.7(Anthropic) for the purposes of language editing assistance, and Microsoft Copilot 365 for the purposes of figure preparation. The author has reviewed and edited the output and takes full responsibility for the content of this publication.

Over the past decades, it has become increasingly clear that macrophages possess remarkable plasticity, allowing them to dynamically adapt their phenotype and function in response to diverse microenvironmental signals. Studies across multiple pathological conditions indicate that transitions between macrophage states rarely occur as discrete switches but rather along a continuum of intermediate phenotypes. This “smeared” distribution of functional traits—including the co-expression of markers traditionally associated with distinct polarization programs, such as CD206 and CD86—poses substantial challenges for both cytometric and molecular characterization, and for the design of narrowly targeted therapies, as their fluid and overlapping profiles may facilitate escape from selective interventions [2,4,5,8,21].

Recent technological advances are reshaping macrophage research through the integration of single-cell and spatial omics, computational modeling, and engineered reprogramming. Single-cell and spatial profiling now resolve macrophage heterogeneity with unprecedented resolution, revealing disease-specific polarization patterns and complex microenvironmental interactions [58,71,143,153,272,273]. Computational approaches integrate multi-scale datasets to predict polarization dynamics, guide drug discovery, and accelerate biomaterial design via machine learning and high-throughput screening platforms. Engineered macrophage systems—such as synthetic receptors, membrane-based nanodecoys, and microbial-cell hybrids—enable precise functional reprogramming for applications in conditions such as cancer, fibrosis, and chronic inflammatory diseases. Together, these advances are shifting the field toward predictive modeling and context-specific immunomodulation, opening new avenues for personalized therapeutic strategies [2,3,4,45].

An important conceptual point emerging from these studies is that the macrophage phenotype most strongly associated with disease progression can be strikingly divergent across distinct disorders and their microenvironments. In many solid cancers, for example, M2-like macrophages exert pro-tumorigenic functions, promoting immune evasion, angiogenesis, and metastasis, whereas similar M2-skewed programs in inflammatory rheumatic and autoimmune diseases often contribute to resolution and tissue repair, with pro-inflammatory M1-like counterparts playing a more pathogenic role. In cancer, this dependence is further illustrated by the metastatic process itself, in which the microenvironment is not simply an extension of the primary tumor but the result of complex co-evolution between tumor clones and organ-specific niches. Macrophage populations in metastatic sites often differ markedly from those in the primary lesion, both in terms of ontogeny and transcriptional programs, underscoring that macrophage function must be interpreted in relation to the specific tissue context [4,15,38,46,96,100,101].

In parallel, the diagnostic and monitoring potential of macrophage-derived soluble markers is gaining increasing attention across multiple disease settings. In renal pathology—particularly in lupus nephritis—an emerging concept is the use of soluble macrophage-related ligands as indicators of disease involvement and progression. Levels of soluble CD163 in serum and urine have been proposed as biomarkers of renal injury, reflecting the burden of CD163+, pro-fibrotic macrophages in advanced lesions [110]. Expanding these panels to include molecules associated with early inflammatory activation, such as CD80 and CD86, may enhance the detection of initial injury phases dominated by M1-like phenotypes, thereby supporting earlier diagnosis and more refined longitudinal assessment.

The same principles extend beyond the kidney to a broad spectrum of inflammation-driven conditions. In osteoarthritis, chronic liver disease, inflammatory bowel disease, and HIV infection, soluble macrophage-related markers—including sCD163, sCD14, and the soluble mannose receptor—have been shown to mirror local macrophage activation and to correlate with disease activity, tissue damage, or prognosis [106,140,141,274,275,276].

Taken together, these observations underscore the rationale for developing macrophage-centered biomarker panels that integrate multiple soluble ligands to improve early detection and longitudinal monitoring of inflammation-mediated tissue injury across diverse clinical contexts.

In the field of cancer immunotherapy, involving macrophages represents a natural extension of the broader strategy of activating the immune system against cancer. Advanced approaches seek not only to enhance T-cell responses—through vaccines, checkpoint blockade, and CAR-T cells—but also to modulate TAMs to reduce local immunosuppression, promote phagocytosis and antigen presentation, and convert immunologically “cold” tumors into inflamed, therapy-responsive lesions. Similarly, while broad TAM depletion may appear conceptually attractive, emerging preclinical and clinical evidence suggests that long-term or non-selective depletion can be detrimental to tissue homeostasis, increase infection risk, and create “ecological niches” that are rapidly occupied by alternative suppressive myeloid populations. The most promising strategies therefore combine (i) selective or temporally restricted targeting of TAM subsets, (ii) reprogramming toward more pro-inflammatory or antigen-presenting phenotypes, and (iii) synergistic integration with T-cell-directed immunotherapies, to recalibrate the entire tumor microenvironment rather than simply removing one cellular component [21,26,41,45,46,47].

Ultimately, advancing the field will require moving beyond rigid, context-specific labels toward a unified, functionally grounded taxonomy of macrophage states that recognizes their continuous phenotypic spectrum. Such a framework, explicitly linking macrophage heterogeneity to disease trajectories, clinical outcomes, and therapeutic vulnerabilities, will be essential to translate macrophage-centered research into reproducible and clinically actionable interventions [2,3,4].

A unifying thread across the pathological contexts examined in this review is the indispensable role of flow cytometry as the operational bridge between molecular biology and clinical phenotyping. Whether monitoring the expansion of hybrid TLR4+M2 monocytes in SSc-ILD peripheral blood [109], tracking the temporal M1-to-M2 transition of macrophages in post-infarction cardiac tissue [142], distinguishing CD206hi versus CD206lo TAM subsets in pancreatic cancer [277], or quantifying CD163+/CD204+ circulating monocytes in PMR and systemic sclerosis [110], flow cytometry provides the resolution and throughput necessary to capture macrophage plasticity in real time and across compartments. Critically, the evolution from conventional multiparameter panels to high-dimensional spectral cytometry and mass cytometry (CyTOF) is now enabling the simultaneous interrogation of surface phenotype, intracellular signaling (phospho-STAT1, phospho-STAT6, NF-kB) [2,28,30,278], metabolic state (BODIPY for lipid loading, MitoTracker for mitochondrial activity), and functional readouts (cytokine secretion by intracellular staining) [144]. This technological convergence positions flow cytometry not merely as a phenotypic descriptor but as a mechanistic tool capable of linking polarization state to disease activity, therapeutic response, and patient stratification. Standardization of macrophage-specific flow cytometry panels—including agreed-upon marker combinations for M1-like (CD80, CD86, HLA-DR, TLR4) and M2-like (CD163, CD204, CD206, CD16) phenotyping across disease contexts—remains an open challenge whose resolution would substantially accelerate both basic research and clinical translation.

## 6. Future Directions

A deeper understanding of the interplay between the immune system and inflammation in diverse pathological contexts will be essential for the development of next-generation therapeutic strategies. In this evolving landscape, macrophages are increasingly recognized as central regulators of the immune–inflammatory axis, owing to their exceptional plasticity, ability to integrate local microenvironmental signals, and capacity to coordinate both inflammatory and tissue-reparative programs [2,3,4,15,46].

Future studies should prioritize the identification and functional characterization of molecular checkpoints that govern macrophage polarization and phenotypic transitions, moving beyond the classical M1/M2 dichotomy toward a more nuanced map of context-specific functional states. Of particular importance will be the dissection of how these macrophage subsets influence disease trajectories in cancer, chronic infections, and cardiovascular disorders, and how their reprogramming may be harnessed to restore immune homeostasis rather than simply suppress inflammation [2,3,4,38,142,261].

Concomitantly, the development of technologies capable of selectively modulating macrophage behavior in vivo—through epigenetic, metabolic, or receptor-targeted interventions—will be critical to achieve precise control of inflammatory responses without impairing host defense. Among the most transformative developments are so-called living drugs, such as CAR-T cells and, more recently, CAR-M therapies, which share the principle of cellular engineering while exhibiting distinct biological and functional properties [3,45].

Advances in single-cell and spatial multi-omics are expected to reveal novel macrophage subsets and functional circuits, opening new therapeutic windows for macrophage-targeted immunotherapies, adoptive cell strategies, and biomaterial-based platforms that exploit their regenerative and immune-orchestrating potential.

Macrophage-based strategies are also emerging as key components in the next generation of therapeutic vaccines, particularly in oncology. One of the major challenges in this field is the induction of durable therapeutic memory, a feature that remains difficult to achieve but is critical for long-term disease control [279].

Together, these converging lines of research underscore a future in which deeper mechanistic insights into inflammation, macrophage biology, and immune engineering will enable increasingly precise and durable therapeutic interventions across multiple disease areas.

## Figures and Tables

**Table 1:** Macrophage polarization phenotypes, mechanisms and significance across pathological microenvironments.

Disease/Context	Dominant Macrophage Phenotype	Key Mechanisms	Clinical/Functional Significance
Breast cancer	M2-like TAMs (CD206+, CD163+, PD-L1+)	CXCL12-driven M1-to-M2 shift; HDAC inhibitors reprogram to M1-like; SOCS2/CCAAT/ CEBPA axis	Immunosuppression; target for HDAC inhibitors and PD-1 blockade
Lung cancer	M2-like/mixed TAMs; interstitial macrophages	Chemotherapy-induced CCL2 increase; coagulation factor X upregulation; reactive myelopoiesis	Pro-metastatic niche formation; chemotherapy paradox
Pancreatic cancer (PDAC)	M2-like TAMs (CD206hi, MARCO+, CD163+)	IGF1/2 chemoresistance; CSF-1R-driven M2 skewing; CCL2/CCR2 myeloid recruitment; Arginase-1-mediated T-cell exclusion	Chemoresistance; CD163/MARCO as prognostic markers
Glioblastoma	Mixed M1/M2 GAMs; TREM2+ immunosuppressive subset	PI3K/AKT-mediated M1-to-M2 transition; CD47-SIRPalpha axis; RT-induced DAMP signaling	Cold TME; resistance to checkpoint blockade; TREM2 as therapeutic target
Hematologic malignancies	M2-like AAMs (CD163+CD206+); CD45highHLA-DR+CD14+CD163high clonal AAMs; M2-skewed LAMs (PD-L1+)	CD47/CD24 ‘don’t-eat-me’ signals blocking phagocytosis; mitochondrial transfer from M2-AAMs to blasts; NAMPT/STAT3/NF-κB M2 skewing in CLL; PI3K–AKT–mTOR axis (FLT3-ITD); HMGB1-driven monocyte-to-TAM differentiation	Chemoresistance in AML; lymphoma progression; CLL myeloid niche
Colorectal cancer	M2-like TAMs; AIEC-educated macrophages	EMILIN-2/TLR4/MyD88/NF-κB M1 polarization; SPP1/TREM2/GPNMB liver-metastasis TAM program; HGF/c-Met extravasation; TAM-derived thymidine phosphorylase and uPA promoting angiogenesis	Liver metastasis niche formation; GPNMB as adverse prognostic marker; pro-tumoral angiogenesis and ECM degradation
Melanoma	M2-like TAMs; immunosuppressive phenotype driven by TGF-β1, IL-10, and tumor-derived exosomes	TGF-β1/IL-10/exosome-mediated macrophage recruitment and M2 skewing; hypoxia and ADM/CD73 shifting arginine metabolism toward M2; VEGF/TGF-β/IL-10 promoting angiogenesis and immune suppression	Immune evasion; resistance to checkpoint immunotherapy
Autoimmune/Rheumatic diseases (RA, SSc, SLE, GCA, PMR)	Mixed M1/M2; hybrid TLR4+M2 monocytes; non-classical CD16+ subsets	NF-kB/STAT in RA synovium; M2c profibrotic in SSc; GM-CSF/M-CSF skewing in GCA; monocyte subset redistribution in PMR	Synovial inflammation; fibrosis; organ damage; macrophage profiling as biomarker
IBD/Skin inflammation	Pro-inflammatory M1-like (active phase); M2-like (resolution)	AIEC macrophage education; TNF-alpha/IL-17 axis; MSC-exosome M2 reprogramming; flavonoid modulation	Mucosal homeostasis breakdown; barrier dysfunction; anti-TNF response
Non-neoplastic lung diseases (asthma, IPF, CLAD) and MAS/HLH	M2/hybrid macrophages; alveolar and hemophagocytic macrophages	CCL18 production; TGF-beta/IL-10 profibrotic axis; ferritin/hemophagocytosis in MAS; TLR4+M2 hybrid phenotype	ILD progression; pulmonary fibrosis; life-threatening cytokine storm in MAS/HLH
Metabolic/Endocrine organ diseases (MI, adipose, kidney, endocrine)	M1-like (injury/inflammation); M2-like (repair/resolution)	Lactate-histone lactylation M1-to-M2 shift; miR-34a/Klf4 M2 suppression in obesity; STAT3-autophagy in DKD; TREM2-LAM in adrenal macrophages	Post-MI remodeling; insulin resistance; diabetic nephropathy; glucocorticoid dysregulation
Infectious diseases	Context-dependent M1/M2; pleural/alveolar macrophages	H. pylori CagA ferroptosis via JAK/STAT1; SARS-CoV-2 efferocytosis disruption; schistosome MST1 activation; CARD9/TREM2 antifungal axis	Host defense vs. immunopathology; chronic inflammation and fibrosis risk
Neurological diseases (stroke, SCI, PD, MS/neurodegeneration)	M1-like microglia/macrophages (acute phase); M2-like repair phase	HMGB1/DAMP-driven M1 activation; TLR4/NF-κB and NLRP3 inflammasome signaling; STING-driven type I IFN promoting late-phase M1 shift in stroke; iron/ferroptosis modulating M1 microglia in MS; glycolytic-NMDAR M1 reprogramming in PD	Neuroinflammation; neurodegeneration; secondary injury; axonal regrowth potential

**Table 2:** Therapeutic strategies targeting macrophage plasticity across disease contexts.

Disease/Therapeutic Context	Strategy/Agent	Macrophage Target/Mechanism	Key Findings
Oncology (breast, lung, glioma, melanoma)	HDAC inhibitors + chemo/PD-1 blockade; liposomal anti-CD137; 2-methoxyestradiol (2ME2)	M2-to-M1 reprogramming of TAMs; CD137 myeloid signaling; STAT3 inhibition via 2ME2	Improved antitumor efficacy in MMTV-PyMT and osteosarcoma models; reduced CD206 and CD163 expression
Oncology (solid tumors, broad)	CAR-macrophages (CAR-M); macrophage-derived EVs; immortalized iBMDMs; exoASO-STAT6/C-EBPbeta	Phagocytosis of tumor cells; EV-mediated reprogramming; M2-to-M1 ASO-mediated silencing	Tumor cell clearance; IL-12 secretion in TME; robust tumor control in orthotopic HCC models
Oncology—checkpoint and phagocytic axes	Anti-CD47/SIRPalpha; anti-CD24/Siglec-10; anti-TREM2; anti-LILRB2 (ILT4)	Removal of ‘don’t-eat-me’ signals; TREM2+ TAM depletion; relief of myeloid suppression	Enhanced phagocytosis; CD8+ T-cell infiltration in cold tumors; synergy with T-cell immunotherapy
Pancreatic cancer (PDAC)	CSF-1R inhibitors; anti-CCL2/CCR2; arginase inhibitor CB-1158; CAR-M targeting CD47 or CSF-1R	Depletion/reprogramming of CD206hi TAMs; CCR2+ monocyte exclusion; arginine restoration for T cells	Improved CD8+ T-cell immunity; sensitization to anti-PD-1; smaller tumors in combination models
Hematologic malignancies (AML, CLL, lymphoma)	NIPA1 depletion; CSF-1R inhibitors (pacritinib, pexidartinib); MIF inhibitors + GM-CSF; CD47-CD20 bispecific antibody	M2-to-M1 shift via IGFBP2/EGFR blockade; TAM depletion in CLL/FL; M1 reprogramming in AML; phagocytosis restoration	Reduced leukemia burden; improved chemosensitivity; superior survival in NHL bispecific models
Autoimmune/Rheumatic diseases (RA, SSc, GCA, SLE, OA)	Metformin; glucocorticoids + MKP-1; TNF inhibitors; nintedanib; mavrilimumab (anti-GM-CSFR); IL-35/IL-38; NR1D1 agonists; TREM2 activators	M1 suppression in synovium; profibrotic M2c prevention in SSc; non-classical monocyte targeting in GCA; MAPK/NF-kB inhibition	Attenuated cartilage damage; reduced vascular inflammation in GCA; improved disease control in SLE and RA
IBD/Skin inflammation (atopic dermatitis)	MSC-derived exosomes; anti-TNF agents; naringenin; diosmetin	MSC-exosome M2 reprogramming; TNF-alpha/IL-17 axis modulation; M1 suppression and M2 promotion by flavonoids	Reduced colitis severity; improved mucosal homeostasis; attenuated skin inflammation in NC/Nga mouse models
Infectious diseases (sepsis, parasites, fungi, viral)	Ophiopogonin C (pyroptosis blockade); MST1 activators; injectable immunoregulatory hydrogels; MIF inhibitors; TREM2 modulators (antifungal)	NLRP3/DDX3X inflammasome inhibition; PPARgamma-CD36 phagocytosis boost; sequential M1-to-M2 biomaterial guidance; antifungal macrophage reprogramming	Reduced organ injury in sepsis; antifibrotic effects in schistosomiasis; improved wound healing; partial restoration of antifungal capacity
Neurological diseases (stroke, SCI, PD)	DAMP-scavenging IL-10 hydrogel; recombinant FGF4 (SCI); TLR4/NF-κB inhibitors; M1 glycolytic/metabolic inhibitors in PD	HMGB1/RAGE neutralization; TLR4/NF-κB blockade in SCI; M1 glycolytic inhibition in PD; FGF4-mediated M2 skewing of microglia/macrophages	Axonal regrowth and motor recovery in SCI models; neuroprotection in PD; attenuation of neuroinflammation in MS and acute CNS injury

## Data Availability

No data were created or analyzed in this study.
